# Structure and biochemical characterization of l-2-hydroxyglutarate dehydrogenase and its role in the pathogenesis of l-2-hydroxyglutaric aciduria

**DOI:** 10.1016/j.jbc.2023.105491

**Published:** 2023-11-22

**Authors:** Jun Yang, Xingchen Chen, Shan Jin, Jianping Ding

**Affiliations:** 1 State Key Laboratory of Molecular Biology, Shanghai Institute of Biochemistry and Cell Biology, Center for Excellence in Molecular Cell Science, Chinese Academy of Sciences, University of Chinese Academy of Sciences, Shanghai, China; 2 School of Life Science and Technology, ShanghaiTech University, Shanghai, China

**Keywords:** 2-hydroxyglutaric aciduria, l-2-hydroxyglutarate dehydrogenase, catalytic mechanism, substrate specificity, protein crystallography

## Abstract

l-2-hydroxyglutarate dehydrogenase (L2HGDH) is a mitochondrial membrane–associated metabolic enzyme, which catalyzes the oxidation of l-2-hydroxyglutarate (l-2-HG) to 2-oxoglutarate (2-OG). Mutations in human L2HGDH lead to abnormal accumulation of l-2-HG, which causes a neurometabolic disorder named l-2-hydroxyglutaric aciduria (l-2-HGA). Here, we report the crystal structures of *Drosophila melanogaste*r L2HGDH (*dm*L2HGDH) in FAD-bound form and in complex with FAD and 2-OG and show that *dm*L2HGDH exhibits high activity and substrate specificity for l-2-HG. *dm*L2HGDH consists of an FAD-binding domain and a substrate-binding domain, and the active site is located at the interface of the two domains with 2-OG binding to the *re*-face of the isoalloxazine moiety of FAD. Mutagenesis and activity assay confirmed the functional roles of key residues involved in the substrate binding and catalytic reaction and showed that most of the mutations of *dm*L2HGDH equivalent to l-2-HGA-associated mutations of human L2HGDH led to complete loss of the activity. The structural and biochemical data together reveal the molecular basis for the substrate specificity and catalytic mechanism of L2HGDH and provide insights into the functional roles of human L2HGDH mutations in the pathogeneses of l-2-HGA.

2-Hydroxyglutarate (2-HG) is a low-abundance metabolite in many mammalian cells produced by a number of metabolic enzymes *via* promiscuous reactions (1, 2, 3). According to the chirality of the C2 atom, 2-HG exists in two enantiomeric forms, namely d-2-HG and l-2-HG. In humans, many enzymes promiscuously catalyze the conversion of 2-oxoglutarate (2-OG) to d-2-HG, including cytoplasmic 3-phosphoglycerate dehydrogenase (4) and mitochondrial hydroxyacid–oxoacid transhydrogenase (5, 6). Besides, the gain-of-function mutants of cytosolic isocitrate dehydrogenase 1 and mitochondrial isocitrate dehydrogenase 2 acquire a neomorphic enzymatic activity, which catalyzes the reduction of 2-OG to d-2-HG as well (7, 8, 9). On the other hand, l-2-HG is produced by mitochondrial and cytoplasmic malate dehydrogenases 1 and 2 and lactate dehydrogenase A under hypoxic or acidic conditions (10, 11, 12). In addition, lactate dehydrogenase C, which is specifically expressed in male and female germ cells and is required for fertility, catalyzes the conversion of a series of 2-oxoacids to 2-hydroxyacids, including the conversion of 2-OG to l-2-HG (13, 14, 15).

In recent years, 2-HG has emerged as an important metabolite associated with cell metabolism (16, 17), epigenetic regulation (18, 19), DNA repair (20, 21, 22), hypoxia signaling (23), and immune response (24, 25, 26, 27). Elevated 2-HG has been observed in a spectrum of malignancies including those in the brain (28, 29), colon (30), kidney (31, 32), breast (33), and pancreas (34). Mechanistically, owing to its structural similarity to 2-OG, 2-HG can competitively inhibit multiple 2-OG/Fe^2+^-dependent dioxygenases, including the Jmjc family of histone demethylases, HIF prolyl hydroxylases, and TET family of DNA dioxygenases, resulting in activation of oncogenic signaling pathways (2, 18, 19). In addition to their roles in tumorigenesis, 2-HG also acts as a regulator in T-cell differentiation and antitumor immunity (24, 25, 26) and exhibits antitumor activity through FTO–m6A–MYC–CEBPA signaling (35). Nevertheless, the exact functional roles of 2-HG and the underlying molecular mechanisms in these biological processes remain elusive.

Under normal cellular conditions, d-2-HG and l-2-HG can be oxidized to 2-OG by d-2-HG dehydrogenase (D2HGDH) and L-2-hydroxyglutarate dehydrogenase (L2HGDH), respectively (1, 3, 36, 37). Mutations of D2HGDH and L2HGDH have been identified in patients of type I d-2-hydroxyglutaric aciduria and l-2-hydroxyglutaric aciduria (l-2-HGA), respectively. d-2-hydroxyglutaric aciduria and L-2-HGA are rare autosomal recessive, neurometabolic diseases that usually begin in infancy or early childhood. These mutations lead to deficient functions of the enzymes and consequently abnormal accumulation of 2-HG in the plasma, urine, and cerebrospinal fluid. In addition, dysfunction of L2HGDH has also been associated with other diseases. For example, defective L2HGDH has been associated with brain tumors (28, 38). Reduced expression of L2HGDH is implicated in the development of renal cancer (31, 39, 40) and colorectal cancer (41). L2HGDH deficiency can lead to impaired adult hippocampal neurogenesis and late-onset neurodegeneration in mouse brains (42, 43). On the other hand, L2HGDH deletion–induced l-2-HG accumulation has been reported to preserve cardiac function under hypoxia conditions in mice, suggesting that L2HGDH inhibition is a potential therapeutic strategy for cardiovascular diseases related to oxidative injury (44). All these findings suggest that L2HGDH plays important roles in a variety of physiological and pathological processes.

L2HGDH belongs to the d-amino acid oxidase (DAAO) family of the FAD-dependent proteins (45). Besides L2HGDH and DAAO, this family also includes enzymes such as glycerophosphate oxidase (GlpO), glycine oxidase, and sarcosine oxidase (45, 46). All members of the family consist of an FAD-binding domain and a substrate-binding domain. Nevertheless, they catalyze different enzymatic reactions for diverse substrates (46, 47, 48, 49). L2HGDH is the only enzyme known to specifically catalyze the oxidation of L-2-HG to 2-OG. L2HGDH exists in bacteria, insects, plants, and animals (1, 50, 51, 52). In higher eukaryotes, it is localized to the mitochondria and associated with mitochondrial membrane (37, 53). So far, the biochemical properties, structure, and catalytic mechanism of L2HGDH are unknown. The functional roles of human L2HGDH mutations identified in l-2-HGA patients remain unclear.

In this work, we performed biochemical and structural studies of *Drosophila melanogaste*r L2HGDH (*dm*L2HGDH). *dm*L2HGDH exhibits high substrate specificity for l-2-HG and has no activity for l-2-HG analogs such as l-malate (L-MAL), l-lactate (L-LAC), and d-2-HG. Crystal structures of *dm*L2HGDH were solved in FAD-bound form and in complex with FAD and 2-OG or SO_4_^2−^. The functional roles of key residues involved in the substrate binding and catalytic reaction were validated by mutagenesis and activity assay. In addition, we also carried out enzymatic activity assay for *dm*L2HGDH mutants containing mutations equivalent to those of human L2HGDH associated with l-2-HGA. The structural and biochemical data together reveal the molecular basis for the high substrate specificity and the catalytic mechanism of L2HGDH and provide insights into the functional roles of human L2HGDH mutations in the pathogeneses of l-2-HGA.

## Results

### *dm*L2HGDH exhibits high substrate specificity for l-2-HG

**Figure 1:**
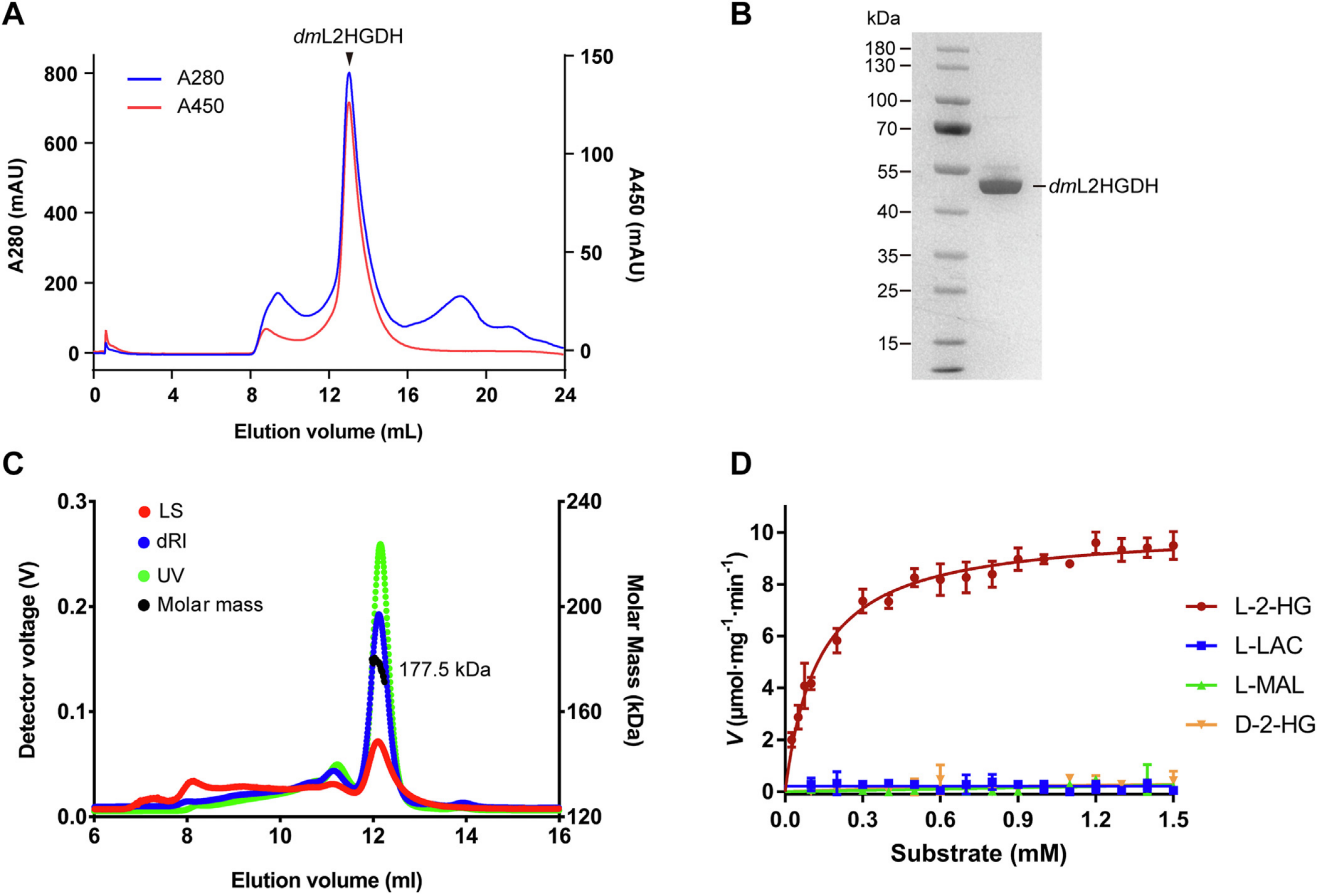
**Biochemical characterization of *dm*L2HGDH.***A,* gel filtration analysis of *dm*L2HGDH. The chromatogram was followed at 280 and 450 nm, which correspond to the characteristic absorptions of protein and FAD, respectively. *B,* SDS-PAGE analysis of purified *dm*L2HGDH. *C,* SEC–MALS analysis of *dm*L2HGDH. Chromatograms show the readings from the light scattering (*red*) at 90°, the refractive index (*blue*), and the UV (*green*) detectors. The *black curve* represents the calculated molecular mass of *dm*L2HGDH (about 178 kDa), corresponding to a tetramer of *dm*L2HGDH. *D,* saturation curves of *dm*L2HGDH for ligands l-2-HG, l-LAC, l-MAL, and d-2-HG. The error bars represent the standard deviations of three independent measurements. *dm*L2HGDH, *Drosophila melanogaster* L2HGDH; l-2-HG, l-2-hydroxyglutaric aciduria; l-LAC, l-lactate; l-MAL, l-malate; SEC–MALS, size-exclusion chromatography–multiangle light scattering.

We initially tried to express human L2HGDH (*hs*L2HGDH) with *Escherichia coli*, insect and mammalian cell expression systems, but failed to obtain sufficient amount of *hs*L2HGDH protein with high purity and stability for biochemical and structural studies. Thus, we turned to several model organisms and eventually succeeded in expressing *dm*L2HGDH in *E. coli* cells with a His_6_-SUMO tag attached to the N terminus and purifying *dm*L2HGDH using a combination of affinity chromatography and gel filtration (Fig. 1*A*). *dm*L2HGDH shares 52% sequence identity with *hs*L2HGDH. As the variable N-terminal region of L2HGDH contains a mitochondrial-targeting peptide (53), the N-terminal 1 to 40 residues of *dm*L2HGDH were removed in the construct. The purified *dm*L2HGDH has high purity, homogeneity, and stability and exists as a tetramer in solution as revealed by SDS-PAGE and size-exclusion chromatography–multiangle light scattering (SEC–MALS) analyses (Fig. 1, *B* and *C*). FAD was tightly bound to the enzyme during the expression and purification processes, resulting in characteristic yellow color of FAD with absorption peak at 450 nm (Figs. 1*A* and S1*A*). By comparing the absorbance spectrum of *dm*L2HGDH-bound FAD and free FAD released from the enzyme after heat denaturation, the extinction coefficient for the *dm*L2HGDH-bound FAD was determined to be 12.51 ± 0.05 mM^−1^ cm^−1^ at 450 nm (Fig. S1*B*). Quantification of the FAD and protein concentrations showed that the FAD occupancy is 0.61 ± 0.02 in the purified *dm*L2HGDH sample.

A previous study showed that rat L2HGDH partially purified from rat liver could specifically catalyze the conversion of l-2-HG to 2-OG with a very weak specific activity of about 1 nmol^.^min^−1.^mg^−1^ but exhibited no activity for l-2-HG analogs l-MAL, l-LAC, and d-2-HG (37). To verify this result, the activity of *dm*L2HGDH using l-2-HG, l-MAL, l-LAC, and d-2-HG as substrate was measured. The results showed that compared with rat L2HGDH, the purified recombinant *dm*L2HGDH catalyzed the conversion of l-2-HG to 2-OG with a much higher specific activity of 9.50 ± 0.31 μmol^.^min^−1.^mg^−1^, a *K*_*M*_ of 0.13 ± 0.008 mM, and a *k*_cat_ of 7.76 ± 0.11 s^−1^ (Fig. 1*D*, Table 1). In addition, the enzymatic activity of *dm*L2HGDH was not affected by the metal ions tested (10 μM Ca^2+^, Mg^2+^, Co^2+^, and Mn^2+^) or EDTA (1 mM) (Fig. S2*A*), indicating that the activity of *dm*L2HGDH is independent of metal ions, which is consistent with the previous report for *hs*L2HGDH (53). However, Zn^2+^ (10 μM) appeared to slightly decrease the activity of *dm*L2HGDH by about 15%; it is possible that Zn^2+^ might affect the stability of the enzyme in the assay condition. Because of the rapid catalytic reaction of l-2-HG by *dm*L2HGDH, the binding of l-2-HG with *dm*L2HGDH was not measurable using isothermal titration calorimetry, surface plasmon resonance, or other methods. On the other hand, *dm*L2HGDH exhibited no activity for l-MAL, l-LAC, and d-2-HG (Fig. 1*D*). Moreover, the enzymatic activity of *dm*L2HGDH for l-2-HG is not affected in the presence of l-LAC or d-2-HG, indicating that these ligands do not bind to *dm*L2HGDH (Fig. S2, *B* and *C*). However, the enzymatic activity of *dm*L2HGDH for l-2-HG is slightly inhibited (<30%) by high concentration (10 mM) of l-MAL, suggesting that l-MAL might have a weak binding to *dm*L2HGDH (Fig. S2*D*). These results together confirm that L2HGDH has high substrate specificity for l-2-HG.

**Table 1:** Specific activity and kinetic parameters of WT *dm*L2HGDH and mutants containing mutations of key residues at the substrate-binding site

*dm*dmL2HGDH	Specific activity (μmol·min^−1^ mg^−1^)	*V*_max_ (μmol·min^−1^ mg^−1^)	*K*_*M*_ (mM)	*k*_cat_ (s^−1^)	*k*_cat_/*K*_*M*_ (s^−1.^ M^−1^)
WT	9.50 ± 0.31	10.15 ± 0.14	0.13 ± 0.008	7.76 ± 0.11	5.97 × 10^4^
R393A	4.77 ± 0.15	5.68 ± 0.19	0.24 ± 0.03	4.34 ± 0.15	1.81 × 10^4^
H302A	0.20 ± 0.10	ND	ND	ND	ND
Y289A	0.14 ± 0.08	ND	ND	ND	ND
H92A	0.13 ± 0.09	ND	ND	ND	ND
S88A	1.07 ± 0.06	1.56 ± 0.17	0.56 ± 0.15	1.19 ± 0.13	2.13 × 10^3^

Abbreviation: ND, not detectable.

### Overall structure of *dm*L2HGDH

Crystallization of *dm*L2HGDH in the absence of any ligands yielded crystals in FAD-bound form (*dm*L2HGDH^FAD^) and in complex with FAD and SO_4_^2−^ (*dm*L2HGDH^FAD+SO4^) in which SO_4_^2−^ was derived from the crystallization solution. Crystals of *dm*L2HGDH in complex with FAD and 2-OG (*dm*L2HGDH^FAD+2-OG^) were obtained by soaking the *dm*L2HGDH^FAD^ crystals with 2-OG. Attempts to obtain the structure of *dm*L2HGDH in complex with l-2-HG by cocrystallization or soaking were unsuccessful because of the rapid catalytic reaction of l-2-HG in solution as revealed by the quickly fading yellow color of the bound FAD of the protein or crystals. In addition, because of the difficulty in expressing and purifying the catalytically defective *dm*L2HGDH mutants containing point mutations at the active site with high quantity, quality, and stability for structural studies, we also failed to obtain the structure of these mutants in complex with l-2-HG.

**Figure 2:**
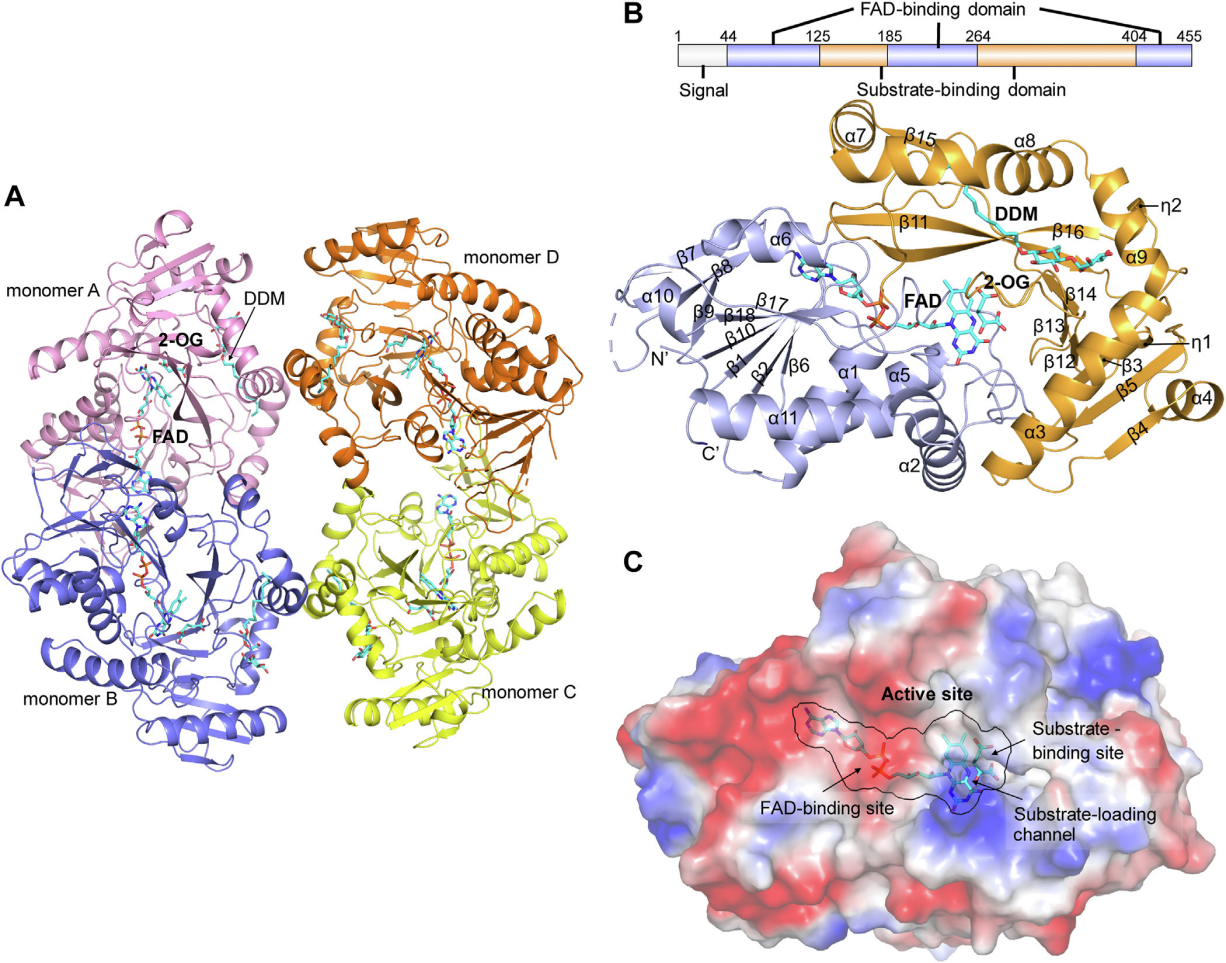
**Crystal structure of *dm*L2HGDH.***A,* assembly of the tetrameric *dm*L2HGDH in complex with FAD and 2-OG. The four monomers (A, B, C and D) of the *dm*L2HGDH tetramer are colored in *pink*, *blue*, *yellow*, and *orange*, respectively. The bound ligands are shown with *stick models* and colored in *cyan*. *B,* overall structure of *dm*L2HGDH in complex with FAD and 2-OG. The FAD-binding domain and the substrate-binding domain are colored in *blue* and *orange*, respectively. The secondary structure elements are labeled. The bound FAD, 2-OG, and DDM are shown with *stick models* and colored in *cyan*. *C,* electrostatic potential surface of *dm*L2HGDH, showing the locations of the FAD-binding site, the substrate-binding site, and the substrate-loading channel. The surface charge distribution is displayed as *white color* for neutral, *red color* for negative, and *blue color* for positive. 2-OG, 2-oxoglutarate; *DDM*, *n*-dodecyl-β-d-maltopyranoside; *dm*L2HGDH, *Drosophila melanogaster* L2HGDH.

Structures of *dm*L2HGDH in FAD-bound form and in complex with FAD and SO_4_^2−^ or 2-OG were determined at resolutions of 2.85 Å, 2.30 Å, and 2.82 Å, respectively (Table 2). Most protein residues are well defined in the electron density map except for a few surface-exposed loops (Table 2). In all structures, there are four *dm*L2HGDH monomers forming a tetramer in the asymmetric unit (Fig. 2*A*). The four monomers share almost identical conformation and are related by noncrystallographic 222 symmetry (Fig. 2*A*). In each monomer, there are an FAD bound at the active site and a detergent molecule *n*-dodecyl-β-d-maltopyranoside (DDM) on a surface groove (Fig. S3, *A* and *B*). The FAD molecule is apparently derived from the expression system. The DDM molecule is derived from the purification buffer with its hydrophilic head lying on the protein surface and the hydrophobic tail pointing to the interior of the protein, which might stabilize the structure. In the *dm*L2HGDH^FAD^ structure, there is no ligand bound at the substrate-binding site; and in the 2-OG- and SO_4_^2−^-bound *dm*L2HGDH structures, there is clearly defined electron density for 2-OG and SO_4_^2−^ at the active site, respectively (Fig. S3, *C*–*E*).

**Table 2:** Summary of diffraction data and structure refinement statistics

	*dm*L2HGDH^FAD^	*dm*L2HGDH^FAD+2-OG^	*dm*L2HGDH^FAD+SO4^
Diffraction data			
Space group	*P*2_1_	*P*2_1_	*C*2
Cell parameters			
*a*, *b*, *c* (Å)	100.1, 103.2, 102.5	100.1, 102.3, 102.6	174.5, 103.1, 122.9
*α*, *β*, *γ* (°)	90, 108.6, 90	90, 108.3, 90	90, 114.6, 90
Resolution (Å)	50.00–2.85 (2.90–2.85)^a^	50.00–2.82 (2.87–2.82)	50.00–2.30 (2.34–2.30)
Observed reflections	306,349	318,863	575,770
Unique reflections	45,678	47,660	86,886
Average redundancy	6.7 (5.7)	6.7 (6.9)	6.6 (4.6)
Average I/σ(I)	15.1 (1.1)	11.0 (1.0)	14.0 (1.3)
Completeness (%)	98.4 (89.9)	99.4 (98.6)	98.4 (98.4)
*R*_merge_ (%)^b^	12.9 (123.4)	17.4 (143.0)	14.5 (95.8)
CC_1/2_	0.99 (0.60)	0.99 (0.53)	0.99 (0.52)
Refinement and structure model			
No. of reflections (*Fo>*0σ(*Fo*))			
Working set	40,050	41,395	84,763
Test set	1991	2005	1988
*R*_work_/*R*_free_^c^	0.244/0.293	0.229/0.278	0.206/0.239
No. of atoms			
Protein	11,634	12,265	12,403
Ligand	352	392	372
Solvent	—	—	539
Missing residues^d^	Chain A: 162–163, 221–223; chain B: 220–224; chain C: 224–228, 413–418; chain D: 220–222	Chain A: 220–223; chain B: 220–223; chain C: 215–216, 220–225, 413–417; chain D: 164–165, 220–223	Chain A: 413–416; chain B: 221–222; chain C: 220–223, 413–417
Wilson *B*-factor (Å^2^)	52.6	53.1	40.6
Average *B*-factor (Å^2^)	49.1	47.6	47.0
Protein	49.3	47.9	47.2
Ligand	42.0	39.4	41.8
Solvent	—	—	47.7
RMSD			
Bond lengths (Å)	0.005	0.002	0.005
Bond angles (^o^)	0.60	0.48	0.97
Ramachandran plot (%)			
Favored	94.6	94.9	96.3
Allowed	5.4	5.1	3.7
Outliers	0	0	0

a Numbers in parentheses represent the statistics for the highest resolution shell.

b *R*_merge_ = ∑_*hkl*_∑_*i*_|*I*_*i*_(*hkl*)−<*I*(*hkl*)>|/∑_*hkl*_∑_*i*_*I*_*i*_(*hkl*).

c *R*-factor = ∑_*hkl*_||*F*_*o*_|−|*F*_*c*_||/∑_*hkl*_|*F*_*o*_|.

d The missing residues have been omitted from the final structure models.

Like other members of the DAAO family, *dm*L2HGDH is composed of the FAD-binding domain and the substrate-binding domain (Fig. 2*B*). The FAD-binding domain adopts a classical “GR2” fold, which is comprised of a six-stranded β-sheet (β1, β2, β6, β10, β17, and β18) and a three-stranded β-sheet (β7–β9) flanked by two α-helices (α6 and α10) on one side and four α-helices (α1, α2, α5, and α11) on the other side. The substrate-binding domain consists of an eight-stranded β-sheet (β3–β5, β12–β14, β11, and β16) surrounded by five α-helices (α3, α4, and α7–α9), and two short 3_10_-helices (η1 and η2) (Fig. 2*B*). A short β-strand (β15) is formed at the interface of monomers A and B (or C and D).

### Structure of the active site

**Figure 3:**
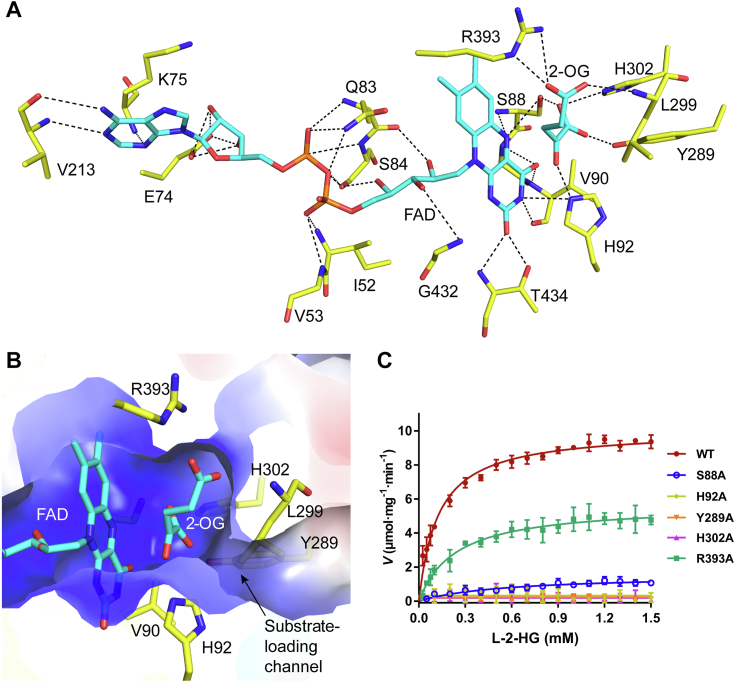
**Structure of the active site of *dm*L2HGDH.***A,* interactions between the FAD and 2-OG molecules with the surrounding residues in the *dm*L2HGDH^FAD+2-OG^ structure. The bound FAD and 2-OG (*cyan*) and the surrounding residues (*yellow*) are shown with *stick models*. The hydrogen bonds are shown with *dashed lines*. *B,* the electrostatic surface of the substrate-binding site in the *dm*L2HGDH^FAD+2-OG^ structure showing a highly positively charged pocket. The bound FAD and 2-OG (*cyan*) and the surrounding residues (*yellow*) are shown as *stick models*. *C,* saturation curves of WT *dm*L2HGDH and the mutants containing point mutations of the key residues interacting with 2-OG. The error bars represent the standard deviations of three independent measurements. 2-OG, 2-oxoglutarate; *dm*L2HGDH, *Drosophila melanogaster* L2HGDH.

The active site of *dm*L2HGDH is located at the interface of the FAD-binding domain and the substrate-binding domain and is composed of residues from both domains (Fig. 2*C*). In all structures, an FAD molecule is bound at the FAD-binding site, which adopts an elongated conformation with the isoalloxazine moiety pointing toward the substrate-binding site and forming extensive hydrophilic and hydrophobic interactions with the surrounding residues (Figs. 3*A* and S4). On the *si*-face of the isoalloxazine moiety, the N5, O4, and N3 atoms form several hydrogen bonds with the main chains and side chains of Ser88 and Val90; on the *re*-face of the isoalloxazine moiety, the N3 atom forms hydrogen bonds with the side chain of His92 (Fig. 3*A*). Sequence alignment shows that most residues involved in the FAD binding are highly conserved in L2HGDH homologs from different species, highlighting their functional role in the FAD binding (Fig. S5).

In the *dm*L2HGDH^FAD+2-OG^ structure, the 2-OG binds to the substrate-binding site, which is located on the *re*-face of the isoalloxazine moiety of FAD and is mainly composed of residues from the substrate-binding domain, including Ser88, His92, Tyr289, His302, Arg393, and Leu299 (Fig. 3*A*). These residues form a positively charged pocket to recognize and bind the acidic moieties of 2-OG (Fig. 3*B*). Specifically, the C1-carboxyl group of 2-OG forms hydrogen bonds with the side chains of Ser88, Tyr289, and His302, and the N5 atom of the isoalloxazine moiety of FAD. The C2-carbonyl group of 2-OG forms a hydrogen bond with the side chain of His92. The C5-carboxyl of 2-OG forms a salt bridge with the side chain of Arg393 and a hydrogen bond with the main chain of Leu299. There is a channel linking the substrate-binding site to the protein surface, which appears to act as the trafficking route for the substrate in and the product out and thus is designated as the “substrate-loading channel” (Fig. 3*B*). In the *dm*L2HGDH^FAD+SO4^ structure, there is a SO_4_^2−^ anion bound at the substrate-binding site, which occupies a similar position as the C1-carboxyl of 2-OG and makes similar interactions with the surrounding residues except for Leu299 (Fig. S6). Structural comparison of *dm*L2HGDH in FAD-bound form and in complex with FAD and 2-OG shows that the 2-OG binding at the active site does not induce notable conformational changes of the residues involved in the FAD and ligand binding (RMSD of ∼0.37 Å for 412 aligned Cα atoms, Fig. S7).

To examine the roles of the key residues interacting with 2-OG in the function of *dm*L2HGDH, we performed mutagenesis and enzymatic activity assay. Alanine substitution of Ser88, Tyr289, and His302, which make hydrogen-bonding interactions with the C1-carboxyl of 2-OG, completely abolished (Y289A and H302A) or significantly impaired (S88A) the activity of *dm*L2HGDH for l-2-HG; mutation of His92, which makes hydrogen-bonding interaction with the C2-carbonyl group of 2-OG, also completely abolished the activity; and mutation of Arg393, which makes hydrogen-bonding interaction with the C5-carboxyl of 2-OG, substantially impaired the activity indicated by decreased specific activity and increased *K*_*M*_ (Fig. 3*C* and Table 1). These results indicate that the key residues involved in the 2-OG binding play important roles in the substrate binding and/or catalytic reaction of *dm*L2HGDH. Sequence alignment shows that these residues are strictly conserved in all L2HGDH homologs from different species, further underscoring their importance in the ligand binding and catalytic reaction (Fig. S5).

### Molecular basis for the high substrate specificity of *dm*L2HGDH for l-2-HG

**Figure 4:**
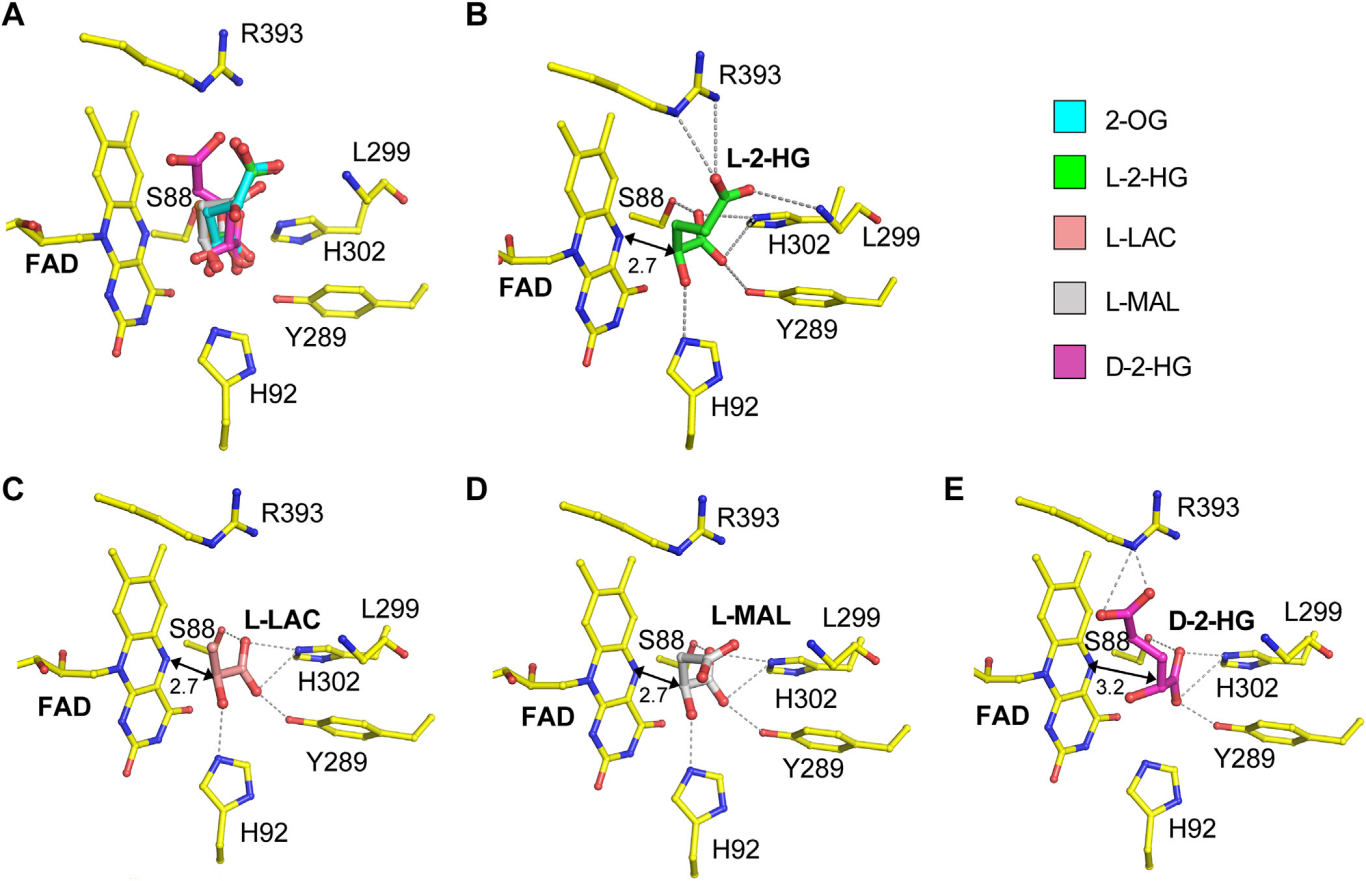
**Molecular basis for the high substrate specificity of *dm*L2HGDH.***A,* superposition of the docked l-2-HG, l-MAL, l-LAC, and d-2-HG at the substrate-binding site based on the position of 2-OG in the *dm*L2HGDH^FAD+2-OG^ structure. *B*–*E,* interactions of l-2-HG (*B*), l-LAC (*C*), l-MAL (*D*), and d-2-HG (*E*) with FAD and the surrounding residues at the substrate-binding site. The potential hydrophilic interactions are shown with *dashed lines*. 2-OG, 2-oxoglutarate; *dm*L2HGDH, *Drosophila melanogaster* L2HGDH; l-2-HG, l-2-hydroxyglutaric aciduria; l-LAC, l-lactate; l-MAL, l-malate.

In order to understand the molecular basis for the high substrate specificity of *dm*L2HGDH for l-2-HG, we docked l-2-HG, l-MAL, l-LAC, and d-2-HG into the active site based on the position and orientation of 2-OG in the *dm*L2HGDH^FAD+2-OG^ structure (Fig. 4*A*). In the structure model of *dm*L2HGDH in complex with l-2-HG, l-2-HG binds to the active site in a similar manner as 2-OG and maintains very similar interactions with the FAD and the surrounding residues without any steric conflicts (Fig. 4*B*). In the structure models of *dm*L2HGDH in complexes with l-LAC and l-MAL, the C1-carboxyl and C2-hydroxyl groups of the ligands adopt similar conformations as those of l-2-HG; however, the smaller functional groups attached to the C3 atom of the ligands are involved in fewer interactions with the surrounding residues compared with l-2-HG (Fig. 4, *C* and *D*), suggesting that the binding of these smaller ligands is weakened or less stable. In addition, it is possible that the binding mode of these smaller ligands might be different from that of 2-OG or l-2-HG because of weakened interactions, and thus the C2-hydroxyl group of these ligands might not be in proper position for deprotonation and the hydride transfer could not take place. These factors might explain in part why *dm*L2HGDH has no activity for l-LAC and l-MAL. In the structure model of *dm*L2HGDH in complex with d-2-HG, to avoid steric conflict of the acetate moiety attached to the C3 atom of d-2-HG with FAD, the C2 atom of d-2-HG is pushed away from the isoalloxazine moiety of FAD. Consequently, the C2 atom of d-2-HG is not in proper position for catalytic reaction (Fig. 4*E*), explaining why *dm*L2HGDH has no activity for d-2-HG. Taken together, these modeling study results provide the molecular basis for the high substrate specificity of L2HGDH for l-2-HG.

### Catalytic mechanism of *dm*L2HGDH

**Figure 5:**
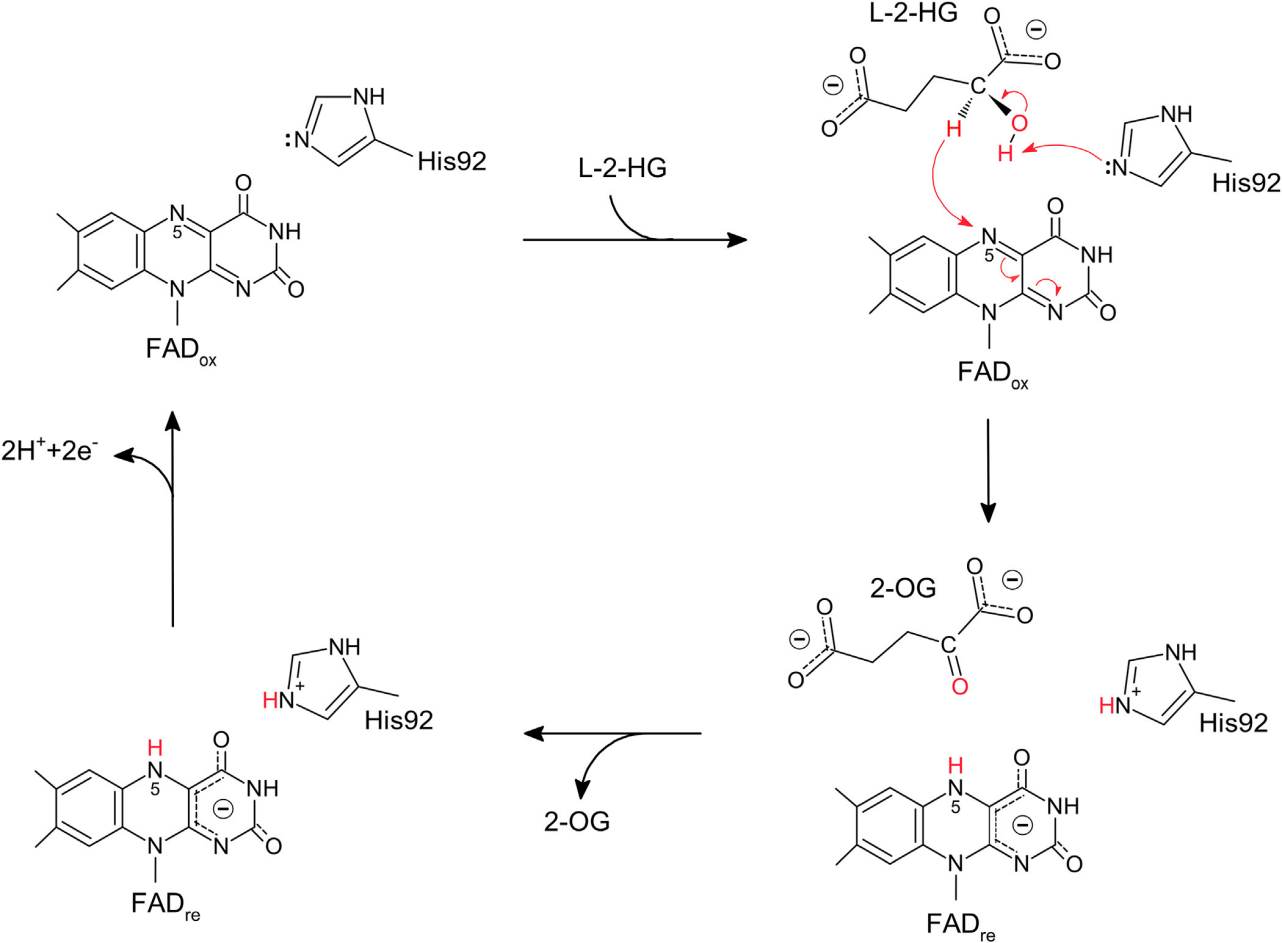
**Proposed catalytic mechanism of *dm*L2HGDH.** His92 acts as the Lewis base to abstract the proton from the C2-hydroxyl group of L-2-HG, and a hydride anion is transferred from the C2 atom of L-2-HG to the N5 atom of FAD, forming a flavin hydroquinone anion and the product 2-OG. Following the release of 2-OG from the active site, the reduced FAD is oxidized by an electron acceptor, and the oxidized FAD then enters the next cycle of catalytic reaction. 2-OG, 2-oxoglutarate; *dm*L2HGDH, *Drosophila melanogaster* L2HGDH; l-2-HG, l-2-hydroxyglutaric aciduria.

GlpO of the DAAO family catalyzes the oxidation of l-2-glycerophosphate (Glp) to dihydroxyacetone phosphate. Previous study has proposed the catalytic mechanism of *Mycoplasma pneumoniae* GlpO (*mp*GlpO) based on structural and biochemical studies (46, 54). *dm*L2HGDH shares a similar overall structure with *mp*GlpO (Protein Data Bank [PDB] code: 4X9M, RMSD = 2.0 Å for 319 align Cα atoms) and binds FAD and 2-OG in a similar manner as *mp*GlpO binding to FAD and Glp (Fig. S8, *A* and *B*). In the *mp*GlpO^FAD+Glp^ structure, His51 at the active site is suggested to function as the Lewis base to abstract a hydrogen from the C2-hydroxyl of Glp (46). In the *dm*L2HGDH^FAD+2-OG^ structure, the equivalent His92 at the active site is the only residue interacting with the C2-carbonyl group of 2-OG, which is also in a proper position to interact with and abstract the hydrogen from the C2-hydroxyl of l-2-HG in the *dm*l-HGDH^FAD+L-2-HG^ structure model, suggesting that His92 may act as the Lewis base in the catalytic reaction. Based on the catalytic mechanism of *mp*GlpO (46), we could propose a similar catalytic mechanism for *dm*L2HGDH (Fig. 5). In this mechanism, His92 acts as the Lewis base to abstract the proton from the C2-hydroxyl group of l-2-HG, and a hydride anion is transferred from the C2 atom to the N5 atom of FAD, leading to the formation of reduced FAD and 2-OG. Then, 2-OG is released from the active site, and the reduced FAD is oxidized by an electron acceptor into the oxidized form before entering the next cycle of reaction. In our *in vitro* activity assay, phenazine methosulfate served as the primary electron acceptor; however, the primary electron acceptor under physiological condition remains unknown.

### Characterization of the L2HGDH mutations associated with l-2-HGA

**Figure 6:**
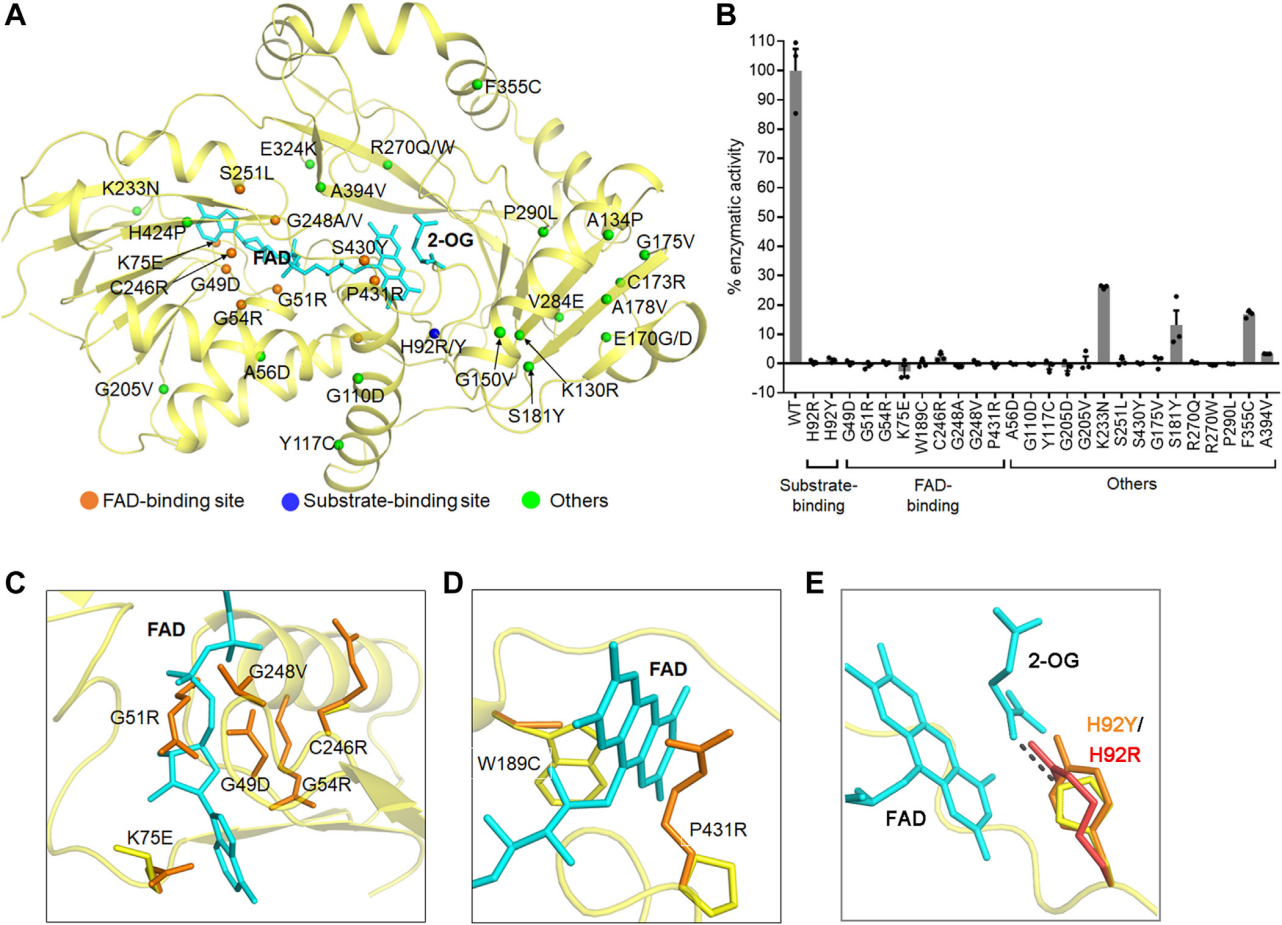
**Functional roles of mutations of *dm*L2HGDH corresponding to****l****-2-HGA-associated *hs*L2HGDH mutations.***A,* locations of mutations in the *dm*L2HGDH^FAD+2-OG^ structure. Mutations at the FAD-binding site, substrate-binding site, and other locations are indicated by *orange*, *blue*, and *green spheres*, respectively. *B,* residual activity of *dm*L2HGDH mutants for l-2-HG are shown as the percentage of the activity of WT *dm*L2HGDH. The error bars represent the standard deviations of three independent measurements. *C,* structure model of mutations at the FAD-binding site, which may induce steric conflicts with the pyrophosphate and adenine moieties of FAD. *D,* structure model of mutations involved in interactions with the isoalloxazine moiety of FAD. *E,* structure model of the H92Y/R mutation at the active site. His92 functions as the catalytic residue. H92Y/R may cause steric conflicts with the substrate binding. l-2-HG, FAD, and mutation residues are shown with *stick models*. *dm*L2HGDH, *Drosophila melanogaster* L2HGDH; *hs*L2HGDH, human (*Homo sapiens*) L2HGDH; l-2-HG, l-2-hydroxyglutarate; L-2-HGA, l-2-hydroxyglutaric aciduria.

Deficient activity of *hs*L2HGDH can lead to l-2-HG accumulation, which has been suggested to be the cause of l-2-HGA. So far, 36 missense mutations and 13 truncating mutations of *hs*L2HGDH have been identified in l-2-HGA patients (55, 56). Truncating mutations usually lead to the absence or the loss of function of the protein and thus are classified as pathogenic. However, the effects of missense mutants of L2HGDH have not been characterized. Although WT *hs*L2HGDH can be expressed in mammalian cells, most of the *hs*L2HGDH mutants containing the disease-associated mutations could not be expressed, suggesting that these mutations affect protein folding and stability and lead to deficient L2HGDH activity. Sequence alignment shows that *hs*L2HGDH and *dm*L2HGDH share high sequence identity and similarity, and the mutated residues associated with l-2-HGA are highly conserved (Fig. S5). Therefore, we mapped the corresponding mutations on the structure of *dm*L2HGDH (Fig. 6*A*) and constructed the maltose-binding protein (MBP)–fused *dm*L2HGDH mutants containing mutations equivalent to the l-2-HGA-associated mutations of *hs*L2HGDH for biochemical characterization. The MBP-fused WT *dm*L2HGDH and mutants could be expressed in *E. coli* cells reasonably well. Notably, although the WT *dm*L2HGDH protein could be purified to high purity, the mutant proteins were mixed to varied extents with bacterial chaperon proteins (HSP70 and SlyD), which are known to facilitate protein folding and increase yield (57) (Fig. S9). This observation again suggests that some of the disease-associated mutations affect protein folding and stability. The percentage of the mutant proteins in total proteins was estimated by SDS-PAGE analysis, and the apparent activity of the mutant proteins was calculated according to the corrected concentrations of the mutant proteins (Fig. S9).

Among the 36 missense *dm*L2HGDH mutants, 10 mutants (H424P, K130R, A134P, G150V, E170G, E170D, C173R, A178V, V284E, and E324K) could not be expressed, suggesting that these mutations severely affect the protein folding and stability. Of the 26 mutants that could be expressed and purified, most mutants caused complete loss of the activity and thus are deemed as pathogenic (Fig. 6*B* and Table S1). According to their locations on the *dm*L2HGDH structure, these mutations can be classified into three groups: mutations at the FAD-binding site, at the substrate-binding site, and at other locations (Fig. 6, *A* and *B*). Mutations of residues forming the FAD-binding site (G49D, G51R, G54R, K75E, W189C, C246R, G248A, G248V, S430Y, and P431R) caused dissociation of FAD from the protein, as indicated by the loss of FAD characteristic absorbance peak at 450 nm (Fig. S10*A*). In particular, Gly49, Gly51, and Gly54 form part of the strictly conserved motif GxGxxG in all DAAO family members; and the absence of side chains of these Gly residues is critical to the binding of the pyrophosphate moiety of FAD (Fig. 6*C*) (45). Lys75 and G248 are involved in the hydrophilic interactions or hydrophobic interactions with FAD; Cys246 is in close proximity to the AMP moiety of FAD; and Trp189 and Pro431 are in close proximity to the isoalloxazine moiety of FAD (Figs. 6*A* and S4). Mutations of these residues would either weaken FAD binding or cause steric clashes with FAD and the surrounding residues (Fig. 6, *C* and *D*). On the other hand, H92R and H92Y are the only mutations located at the substrate-binding site. These mutants retained the FAD-binding ability as indicated by the presence of FAD characteristic absorbance peak at 450 nm (Fig. S10*B*). However, as His92 is the key residue for interaction with the C2-carboxyl group and deprotonation of the substrate, substitution of His92 with tyrosine or arginine with longer side chain may cause steric conflicts with the substrate and thus impair the substrate binding and/or catalytic reaction, leading to complete loss of the activity (Fig. 6*E*). In addition, mutations A56D, G110D, Y117C, G175V, G205D, G205V, S251L, R270Q, R270W, and P290L are not directly involved in direct interactions with FAD or substrate but also caused complete loss of the activity (Figs. 6*A* and S10*C*). It is possible that these residues might play a structural role in the protein folding, and their mutations affect the stability of the enzyme. There are four mutants (K233N, F355C, S181Y, and A394V; Fig. S10*D*), which exhibited a residual enzymatic activity of 26.2%, 16.9%, 13.2%, and 4.6% compared with the WT protein, respectively. The pathogenicity of all 36 missense mutations was predicted using the PolyPhen-2 server (58). Only the K233N mutation was predicted to be benign, and other mutations were appraised as damaging to varied degree (Table S1). The prediction results are in agreement with our biochemical data of the *dm*L2HGDH mutants (Table S1).

## Discussion

D2HGDH and L2HGDH are responsible for catalyzing the oxidation of d-2-HG and l-2-HG into 2-OG, respectively. Although both L2HGDH and D2HGDH are FAD-dependent enzymes, they belong to different protein families with a low sequence identity (∼13%) and structure similarity. D2HGDH contains an FAD-binding domain with a “PCMH” type fold, whereas L2HGDH contains an FAD-binding domain with a “GR2” fold (45, 59). The substrate-binding site of *dm*L2HGDH is located on the *re*-face of the isoalloxazine moiety of FAD, whereas the substrate-binding site of *hs*D2HGDH (PDB code: 6LPP) (59) is located on the *si*-face of the isoalloxazine moiety of FAD. Thus, the two enzymes exhibit distinct substrate stereoselectivity by dictating the spatial orientation and arrangement of FAD and the substrate.

*dm*L2HGDH exists as a tetramer in both solution and crystal structure; however, the biologically relevant oligomeric form is unclear. Analysis of the buried surface areas between the four monomers (designated as monomers A, B, C, and D, Fig. S11*A*) shows that the buried surface areas at both the A–B and A–D dimer interfaces are about 400 to 450 Å^2^, each accounting for only 2% of the total solvent-accessible surface area of a monomer (about 20,000 Å^2^). The interactions at the A–B (or C–D) interface are stronger than those at the A–D (or B–C) interface. The A–D (or B–C) interface is mediated by several hydrophobic interactions between two α7-helices (Fig. S11*B*). On the other hand, the A–B (or C–D) interface is mediated by both hydrophilic and hydrophobic interactions as well as a disulfide bond formed by two Cys77 residues (Fig. S11, *C* and *D*). Substitution of Cys77 with alanine did not affect the tetrameric state of *dm*L2HGDH in solution but substantially reduced the enzymatic activity, indicating that there might be cooperative effect between monomers A and B (Fig. S11*E*). These results suggest that the dimer formed by monomers A and B (or C and D) might be biologically relevant. Nevertheless, the residues involved in the interactions at the A–B and A–D interfaces are not conserved among L2HGDH homologs (Fig. S11*F*). It is unclear whether the cooperativity between monomers is applicable to *hs*L2HGDH, and the impact of the deleted N-terminal region on the formation of tetramer is also unknown.

*dm*L2HGDH belongs to the FAD-dependent DAAO family, all members of which contain an FAD-binding domain with a conserved “GR2” protein fold. The search of structurally similar proteins in the PDB on the DALI server (60) identified *Bordetella pertussis* protein Bp3253 (PDB code: 3DME, Z-score = 42.2) and *mp*GlpO (PDB code: 4X9M, Z-score = 40.6) as the most similar proteins. Bp3253 shares 33.1% sequence identity with *dm*L2HGDH with unknown function. Structural comparison shows that the structures of *dm*L2HGDH, *hs*DAAO, *mp*GlpO, and Bp3253 share a highly conserved FAD-binding domain with the bound FAD adopting a similar extended conformation; however, the structures of the substrate-binding domains show greater variations (Fig. S12, *A*–*D*). All the residues involved in the substrate binding are strictly conserved in Bp3253, suggesting that Bp3253 might have a similar substrate specificity as L2HGDH (Fig. S12*E*). However, only the residues involved in the interactions with the C1-carboxyl and C2-hydroxyl groups (Ser88, His92, Tyr289, and Arg393 in *dm*L2HGDH) are conserved in *mp*GlpO, and only the residues involved in the interactions with the C1-carboxyl and C5-carboxyl groups (Ser88, Tyr289, Leu299, and Arg39 in *dm*L2HGDH) are conserved in *hs*DAAO (Fig. S12*E*). These sequence and structural differences might contribute to their distinct substrate specificities and different catalytic reactions.

So far, L2HGDH is the only enzyme in humans reported to specifically act on l-2-HG. The removal of l-2-HG is thought to be evolutionarily conserved and functionally important in both prokaryotes and eukaryotes (2). Previously, a series of missense mutations has been identified in l-2-HGA patients with unclear functional roles. In this work, the biochemical and structural analysis confirmed that these mutations affect residues that are directly or indirectly involved in FAD and substrate binding, catalysis, and/or protein stability, leading to loss or substantial reduction of the L2HGDH activity. This study provides a better understanding of the pathogenesis of l-2-HGA and other diseases associated with L2HGDH deficiency. The potential of L2HGDH to serve as a diagnostic marker or therapeutic target in l-2-HG-driven diseases is worthy of further investigation.

## Experimental procedures

### Cloning, expression, and purification

The gene encoding the full-length *dm*L2HGDH was synthesized by Sangon Biotech. The *dmL2HGDH* gene fragment with the N-terminal 1 to 40 residues truncated (residues 41–455) was cloned into the pET-28a expression vector (Novagen) with an N-terminal His_6_-SUMO tag. The N-terminal 1 to 40 residues of *dm*L2HGDH are highly variable among different species and are deemed as a mitochondrial-targeting peptide and thus were removed in the construct. The truncated *dm*L2HGDH protein was more stable than the full-length protein. The plasmid was transformed into *E. coli* BL21 Codon-Plus (DE3)-RIPL strain (Weidi Biotech), and the transformed cells were grown in LB medium containing 0.05 mg/ml kanamycin at 37 °C until an absorbance reached 0.8 at 600 nm and then induced with 0.2 mM IPTG at 16 °C overnight. The bacterial cells were collected and then lysed by a high-pressure cell disrupter in buffer A (25 mM Hepes, pH 7.5, 200 mM NaCl, and 5% glycerol) supplemented with 1 mM PMSF, 1% Triton X-100, and 10 mM imidazole followed by centrifugation at 18,000 rpm for 1 h at 4 °C. The target protein was purified by affinity chromatography using an nickel–nitrilotriacetic acid (Ni–NTA) column (Qiagen) with buffer A supplemented with 40 mM imidazole and 0.018% (w/v) DDM (Anatrace) as wash buffer and 250 mM imidazole and 0.018% (w/v) DDM as elution buffer. The His_6_-SUMO tag was hydrolyzed by ULP1 during dialysis against buffer A and then removed by rebinding to the Ni–NTA column. The flow-through fractions containing the *dm*L2HGDH protein were concentrated in 30 kDa molecular mass cutoff concentrators (Amicon), and the protein was further purified by Superdex 200 10/300 column (Cytiva) in buffer A. Constructs of the activity-deficient *dm*L2HGDH mutants (S88A, H92A, Y289A, H302A, and R393A) were generated using the QuikChange Site-Directed Mutagenesis kit (StrataGene). Expression and purification of the mutants were the same as the WT protein. The purified proteins were of high purity, homogeneity, and stability as evaluated by SDS-PAGE and SEC–MALS analyses.

The *dm*L2HGDH mutants containing point mutations corresponding to the disease-associated mutations of *hs*L2HGDH were also constructed using the QuikChange Site-Directed Mutagenesis kit. The DNA fragments encoding WT and mutant *dm*L2HGDH were cloned into the pRSFDuet-1 vector (Novagen) with a His_6_ tag and an MBP protein attached to the N terminus of *dm*L2HGDH protein, resulting in the His_6_-MBP-*dm*L2HGDH constructs. The plasmids were transformed into *E. coli* BL21 Codon-Plus (DE3)-RIPL strain for protein expression. The His_6_-MBP-fused proteins were purified by Ni–NTA affinity chromatography using the previously described wash and elution buffers. The total protein concentrations in the eluted samples from Ni–NTA affinity chromatography were determined by Bradford assay using a bovine serum albumin standard curve. As the mutant proteins were always mixed to varied extents with bacterial chaperon proteins and trace amounts of some other impurities, the samples of MBP-*dm*L2HGDH mutants were subjected to SDS-PAGE analysis on a Bio-Rad Gel Doc EZ Imager to estimate the percentage of the mutant proteins in total proteins. The major bands of contaminants at 70 and 25 kDa were excised from the SDS-PAGE gel, and the compositions of these impurities were analyzed by mass spectrometry at the National Facility for Protein Science in Shanghai. The apparent activity of the mutant proteins was calculated according to the corrected concentrations of the mutant proteins.

### SEC–MALS analysis

The molar mass of the purified protein was analyzed by SEC–MALS on an Agilent 1260 Infinity Isocratic Liquid Chromatography System (Agilent) incorporated with a Wyatt Dawn Heleos II Multi-Angle Light Scattering Detector and a Wyatt Optilab T-rEX Refractive Index Detector (Wyatt Technology). Protein solution (2 mg/ml) was injected into a Superdex 200 10/300 column with a mobile phase containing 20 mM Hepes (pH 7.4) and 200 mM NaCl at a flow rate of 0.4 ml/min. The eluate was monitored with three detectors for UV absorption, light scattering, and refractive index. The UV and refractive index detectors were used to quantify the protein concentration in two orthologous ways; and the light scattering detector was used to determine the protein molar mass. The data were analyzed using the ASTRA software (Wyatt Technology) to determine the molar mass of the protein.

### Enzymatic activity assay

The enzymatic activity of *dm*L2HGDH was determined by monitoring the reduction of 2,6-dichloroindophenol (DCIP) spectrophotometrically at 600 nm in a reaction mixture (200 μl) containing 50 mM Hepes (pH 7.5), 0.5 μg enzyme, 200 μM phenazine methosulfate, 100 μM DCIP, and varied concentrations of different ligands (l-2-HG, l-MAL, l-LAC, and d-2-HG) incubated at room temperature (about 25 °C). The reaction was initiated by addition of the ligand. The extinction coefficient of DCIP is 22 cm^−1^ mM^−1^. The activity is defined as the micromole of DCIP reduced per minute per milligram of enzyme (μmol^.^min^−1.^mg^−1^). The specific activity of WT and mutant *dm*L2HGDH was determined at the standard conditions with a fixed substrate concentration (1.5 mM). The inhibition assay of l-MAL, l-LAC, or d-2-HG on the activity of *dm*L2HGDH was assessed by incubating *dm*L2HGDH with varied concentrations of ligand (0, 2, and 10 mM) and initiated by addition of substrate l-2-HG (1.5 mM). The kinetic data were measured with varied concentrations of substrate (0–1.5 mM). The kinetic parameters (*V*_max_, *K*_*M*_, and *k*_cat_) were obtained by fitting the kinetic data into the Michaelis–Menten equation “*V* = *V*_max_∗[S]/(*K*_*M*_ + [S])” using program GraphPad Prism (GraphPad Software). All experiments were performed in triplicates using distinct samples.

The concentration of protein in the purified *dm*L2HGDH sample was determined using the extinction coefficient of *dm*L2HGDH at 280 nm (47.33 mM^−1^ cm^−1^). The concentration and extinction coefficient of FAD bound to the *dm*L2HGDH protein was determined as follows. First, the absorbance at 450 nm was measured for FAD-bound *dm*L2HGDH. Then, the sample was heated at 100 °C for 5 min for protein denaturation. During denaturation, FAD was released from the enzyme, and the molar extinction coefficient of FAD was altered. The denatured protein was removed by centrifugation after cooling, and the absorbance at 450 value of the supernatant containing free FAD was measured. Concentration of free FAD was determined with an extinction coefficient of 11.3 mM^−1^ cm^−1^ at 450 nm (61). By comparing the absorbances of the enzyme-bound FAD and released FAD (Fig. S1*B*), the extinction coefficient for the enzyme-bound FAD was determined to be 12.51 ± 0.05 mM^−1^ cm^−1^ at 450 nm. The FAD occupancy was determined to be 0.61 ± 0.02 for the purified WT *dm*L2HGDH protein. The specific activity and kinetic parameters of *dm*L2HGDH were corrected according to the concentration of active enzyme in the reaction solution calculated based on the FAD occupancy.

### Crystallization, diffraction data collection, and structure determination

Crystallization of *dm*L2HGDH was performed using the hanging-drop vapor diffusion method at 16 °C by mixing equal volume of the protein solution (8 mg/ml) and the reservoir solution. Crystals of *dm*L2HGDH in FAD-bound form were grown in drops containing 0.2 M magnesium formate and 18% (w/v) PEG 3350. Crystals of *dm*L2HGDH in complex with FAD and SO_4_^2−^ were grown in drops containing 0.2 M lithium sulfate, 0.1 M Bis–Tris (pH 5.9), and 23% (w/v) PEG 3350. Crystals of *dm*L2HGDH in complex with FAD and 2-OG were obtained by soaking the FAD-bound *dm*L2HGDH crystals in the crystallization solution supplemented with 40 mM 2-OG for 10 min. For diffraction data collection, the crystals were cryoprotected using the reservoir solution supplemented with 20% glycerol and then flash-cooled in liquid _nitrogen_. Diffraction data were collected at 100 K at BL18U1 and BL19U1 of the National Facility for Protein Science in Shanghai, China and processed with HKL3000 (62). Statistics of the diffraction data are summarized in Table 2.

The structure of *dm*L2HGDH in complex with FAD and SO_4_^2−^ was solved by the molecular replacement method implemented in Phenix using the AlphaFold predicted structure model of *dm*L2HGDH as the search model. The structures of *dm*L2HGDH in complex with FAD and in complex with FAD and 2-OG were solved by the molecular replacement method using the FAD and SO_4_^2−^-bound *dm*L2HGDH structure as the search model. Structure refinement was carried out using Phenix and Refmac5 (63, 64). Model building was performed using Coot (MRC Laboratory of Molecular Biology) (65). Structure analysis was performed with programs in the CCP4 suite (66). Structure analysis was performed with PyMOL (Schrödinger, LLC) (67) and LigPlot^+^ (European Bioinformatics Institute) (68). Statistics of the structure refinement and the quality of final structure models are also summarized in Table 2.

## Data availability

Atomic coordinates and structure factors of the *dm*L2HGHD^FAD^, *dm*L2HGHD^FAD+2-OG^, and *dm*L2HGHD^FAD+SO4^ structures have been deposited with the PDB under accession codes 8W75, 8W78, and 8W7F, respectively.

## Supporting information

This article contains supporting information (37, 53, 55, 58, 68, 69, 70, 71, 72, 73, 74, 75, 76, 77, 78, 79, 80, 81, 82, 83, 84, 85).

## Conflict of interest

The authors declare that they have no conflicts of interest with the contents of this article.
