# Supplementary material for: Structure and biochemical characterization of l-2-hydroxyglutarate dehydrogenase and its role in the pathogenesis of l-2-hydroxyglutaric aciduria
Source: J Biol Chem. 2023 Nov 22;300(1):105491. doi: 10.1016/j.jbc.2023.105491 (PMC10726252; doi:10.1016/j.jbc.2023.105491)
Supplement: Supporting Figures S1–S12 and Table S1 [file mmc1.pdf]

Supporting Information

Structure and biochemical characterization of L-2-hydroxyglutarate dehydrogenase and its role in the pathogenesis of L-2-hydroxyglutaric aciduria

Jun Yang[1,$], Xingchen Chen[1,$], Shan Jin[1], and Jianping Ding[1,2,*]

[1] State Key Laboratory of Molecular Biology, Shanghai Institute of Biochemistry and Cell Biology, Center for Excellence in Molecular Cell Science, University of Chinese Academy of Sciences, Chinese Academy of Sciences, 320 Yue-Yang Road, Shanghai 200031, China

[2] School of Life Science and Technology, ShanghaiTech University, 393 Huaxia Zhong Road, Shanghai 201210, China

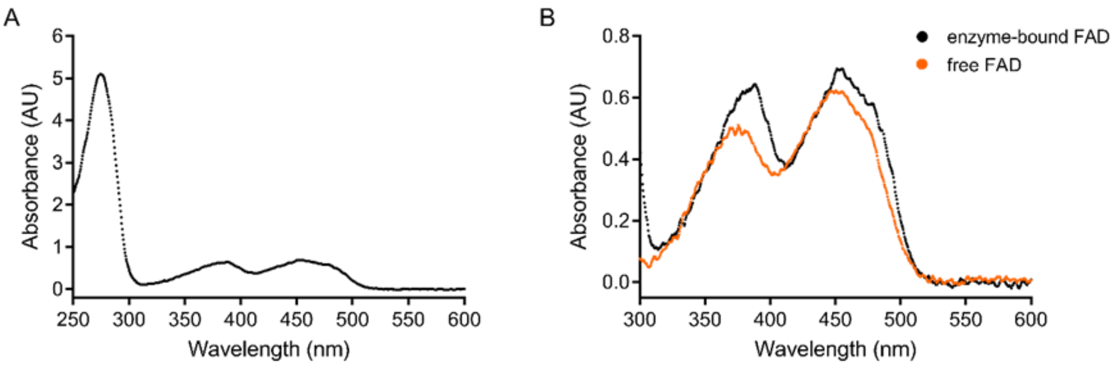

Figure S1. UV-Vis absorbance spectra of FAD-bound *dm*L2HGDH and released FAD. (A) UV-Vis spectrum (250 – 600 nm) of the purified FAD-bound *dm*L2HGDH sample, showing characteristic absorbance peaks for protein (280 nm) and FAD (450 nm). (B) Zoom-in view of the UV-Vis spectra (300 – 600 nm) of the enzyme-bound FAD (black) and free FAD released from *dm*L2HGDH upon heat denaturation (orange). The extinction coefficient of the enzyme-bound FAD was determined to be 12.55 $mM^{-1}\cdot cm^{-1}$.

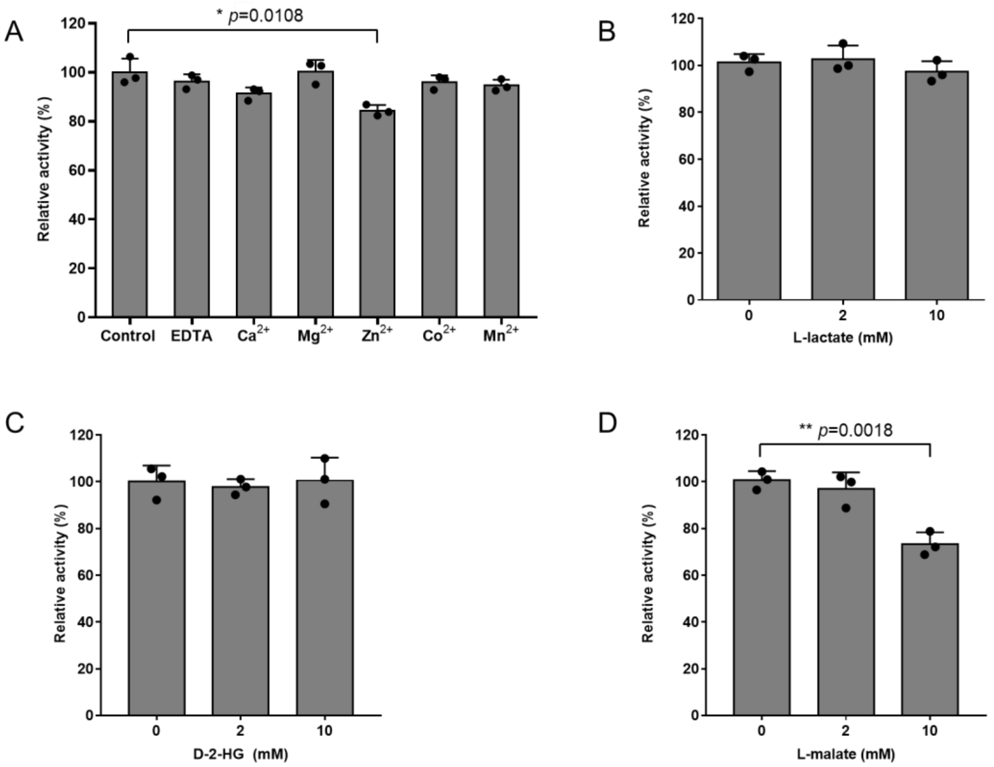

Figure S2. Effects of metal ions, EDTA and substrate analogs on the activity of *dm*L2HGDH for L-2-HG. (A) Relative activity of *dm*L2HGDH in the absence (Control, 100%) and presence of EDTA (1 mM) or different metal ions (10 μM). (B-D) Relative activity of *dm*L2HGDH in the presence of varied concentrations of L-lactate (B), D-2-HG (C), and L-malate (D). The error bars represent the standard deviations of three independent experiments. *P* values were calculated using two-sided unpaired t test.

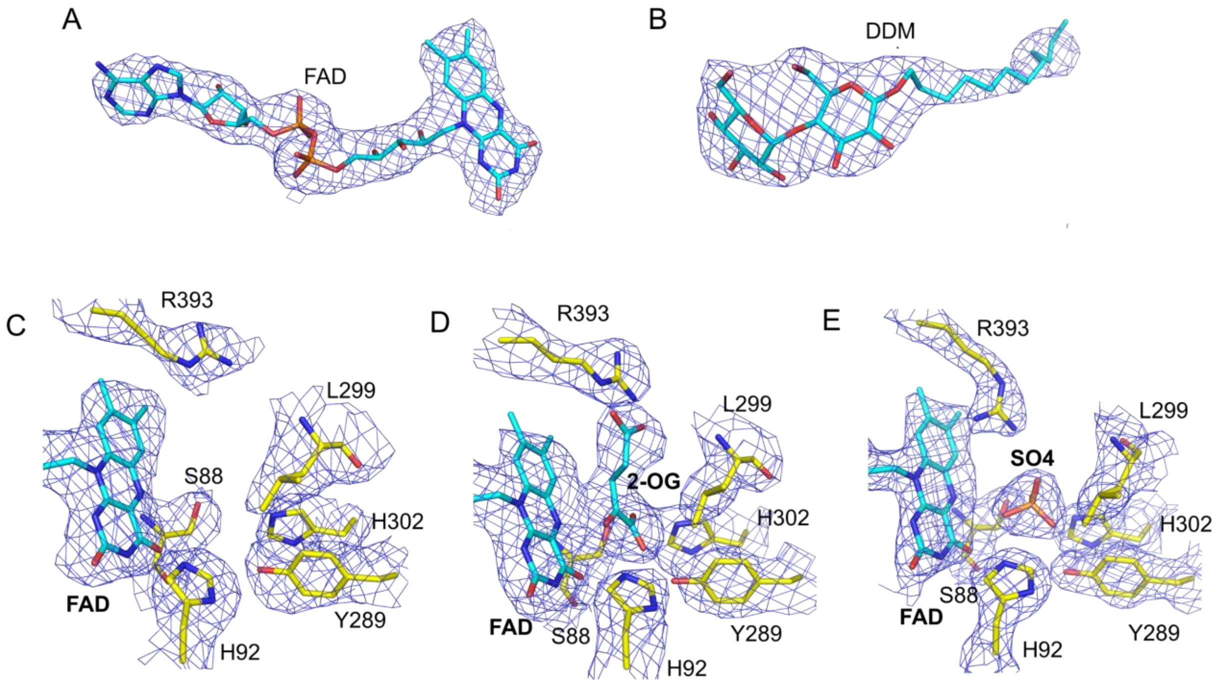

Figure S3. Representative simulated annealing composite 2*Fo*-*F*c omit maps (1.0 σ contour level) in the *dm*L2HGDH structures. (A) The FAD molecule in the *dm*L2HGDH^{FAD} structure. **(B)** The DDM molecule in the *dm*L2HGDH^{FAD} structure. **(C)** The active site in the *dm*L2HGDH^{FAD} structure. **(D)** The active site in the *dm*L2HGDH$^{FAD+2\text{-}OG}$ structure. **(E)** The active site in the *dm*L2HGDH$^{FAD+SO4}$ structure. The ligands and surrounding residues are shown with stick models.

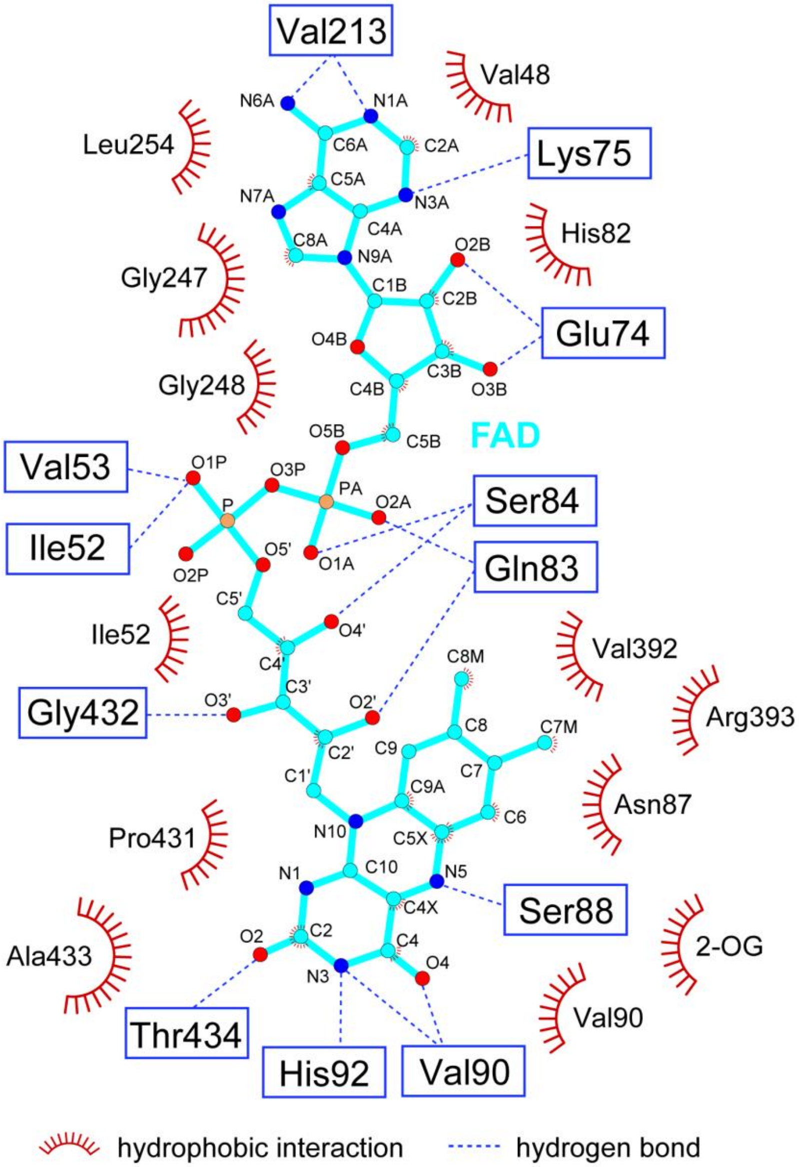

Figure S4. Schematic diagram showing the interactions of FAD with the surrounding residues in the *dm*L2HGDH$^{FAD+2\text{-}OG}$ structure. Hydrogen bonds are shown with blue dashed lines and hydrophobic interactions are represented as red eyelashes around the interacting residues and FAD atoms. The residues involved in hydrogen-bonding interactions with FAD are indicated with blue rectangles. The diagram was generated by LigPlot (66).

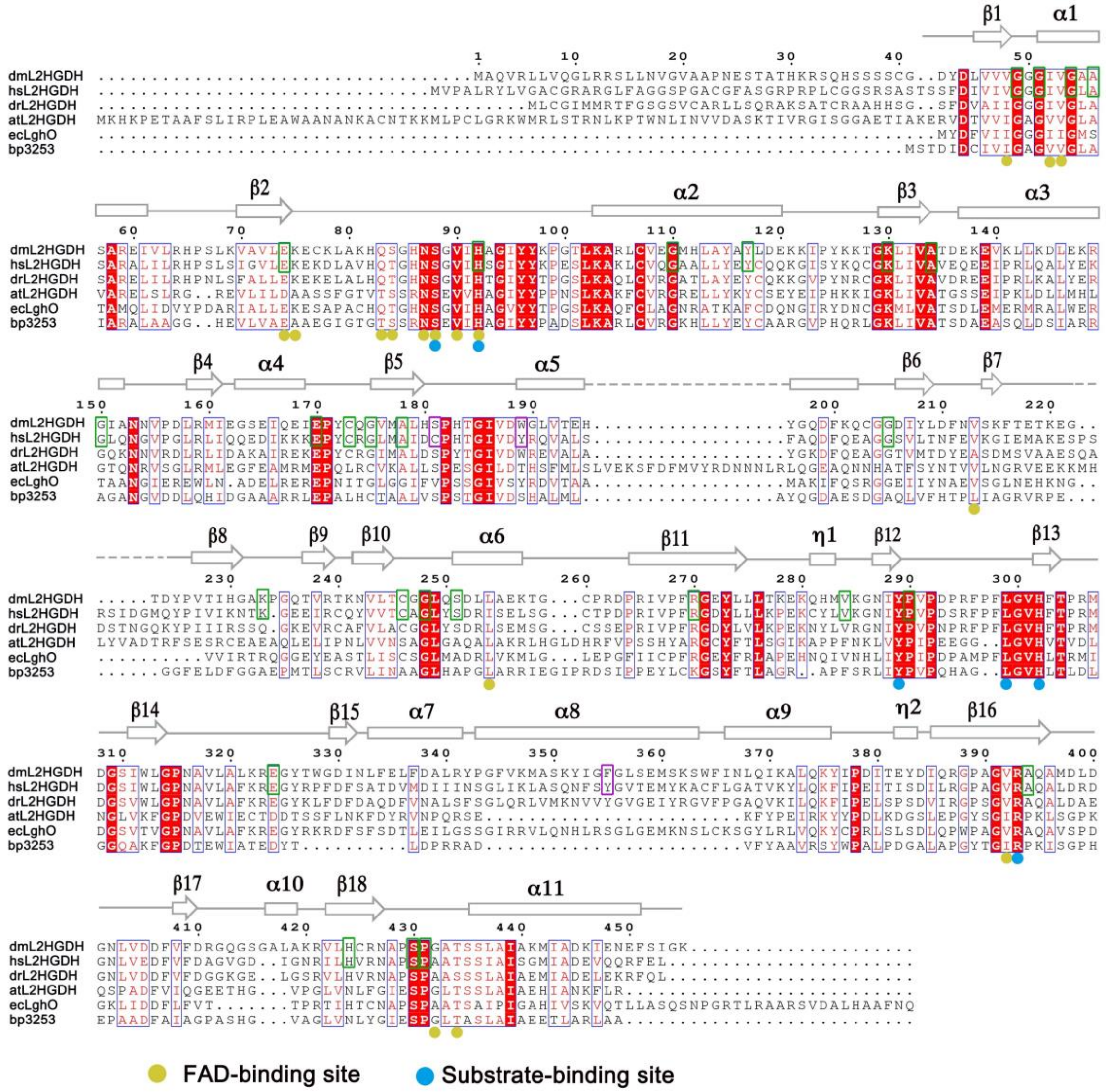

Figure S5. Sequence alignment of *dm*L2HGDH homologs from different species. The secondary structures of *dm*L2HGDH are placed on the top of the sequence alignment. The conserved residues are highlighted in open red boxes and the strictly conserved residues are highlighted in shaded red boxes. The key residues of *dm*L2HGDH involved in the binding of 2-OG and FAD are indicated with cyan and yellow spheres, respectively. Residues whose mutations are associated with L-2-HGA are either strictly or highly conserved in *dm*L2HGDH and *hs*L2HGDH, and are marked with open green and purple boxes, respectively. Abbreviations: hs, *Homo sapiens*; dr, *Danio rerio*; dm, *Drosophila melanogaster*; at, *Arabidopsis thaliana*; ec, *Escherichia coli*; and bp, *Bordetella pertussis*.

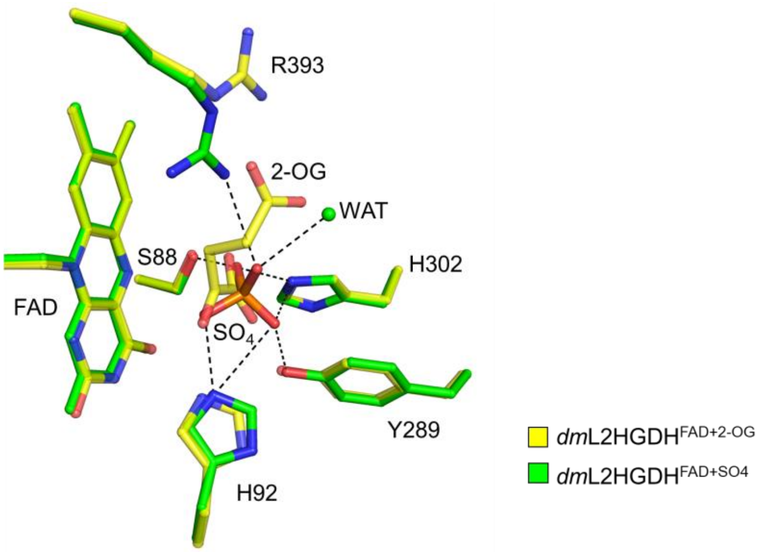

Figure S6. Superposition of the substrate-binding sites in the *dm*L2HGDH$^{FAD+2-OG}$ (yellow) and *dm*L2HGDH$^{FAD+SO4}$ (green) structures. The isoalloxazine moiety of FAD, 2-OG, SO_4^{2-} and the active site residues are shown with stick models, and water molecule is shown with a green sphere. The hydrogen bonds between SO_4^{2-} and the surrounding residues in the *dm*L2HGDH$^{FAD+SO4}$ structure are indicated with dashed lines.

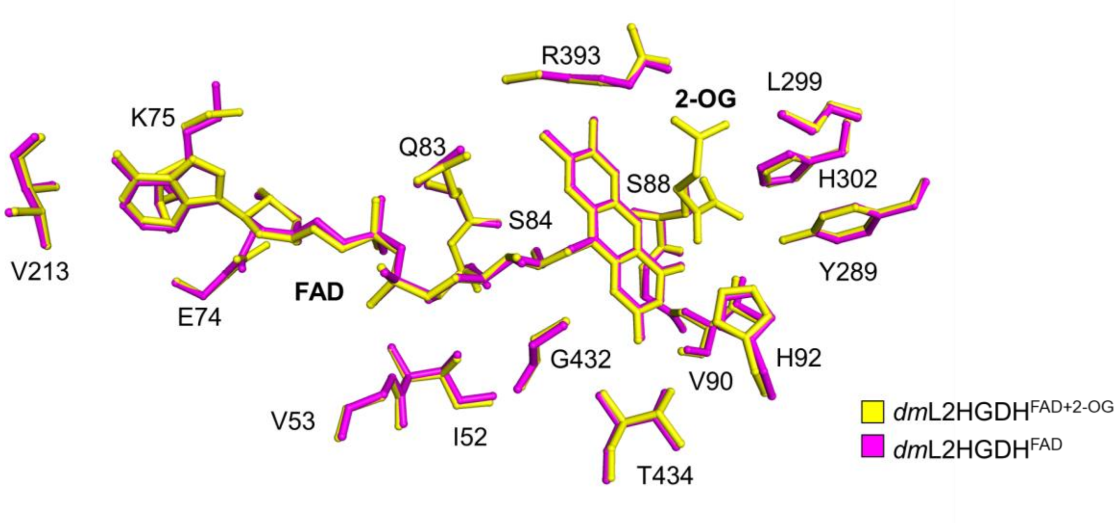

Figure S7. Superposition of the active sites in the *dm*L2HGDH^{FAD} and *dm*L2HGDH$^{FAD+2\text{-}OG}$ structures. The FAD, 2-OG and surrounding residues are shown with stick models.

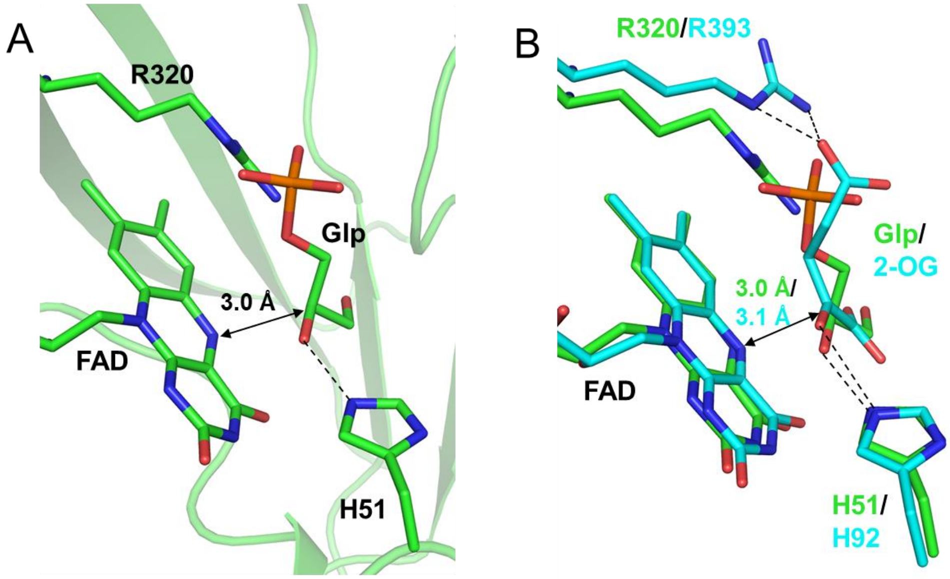

Figure S8. Structure comparison of *mp*GlpO in complex with Glp and *dm*L2HGDH in complex with 2-OG. (A) The active site of *mp*GlpO (PDB 4X9M) showing the positions and orientations of the bound substrate and key residues His51 and Arg320. (B) Structure comparison of the active sites of *mp*GlpO and *dm*L2HGDH. The ligands (2-OG and Glp) bind to the active site of the respective enzyme in similar position and orientation. The hydrogen bonds are shown as dashed lines, and the distances between the C2 atom of the ligands and the N5 atom of FAD are indicated.

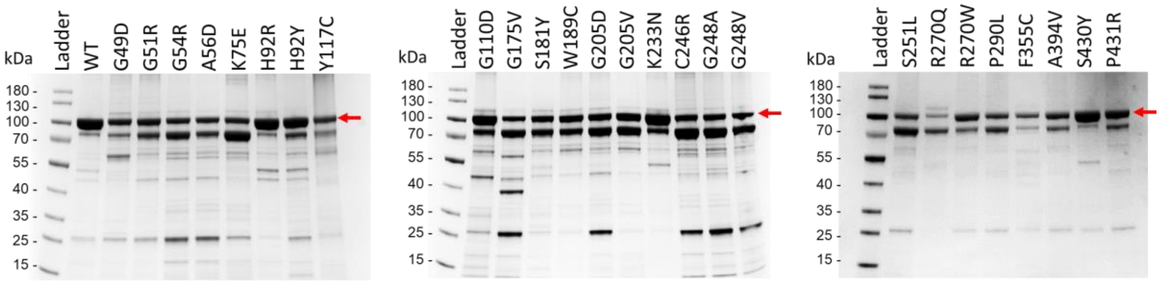

Figure S9. Purification of *dm*L2HGDH mutants containing mutations corresponding to L-2-HGA-associated *hs*L2HGDH mutations. Wild-type (WT) *dm*L2HGDH and *dm*L2HGDH mutants were expressed as N-terminal His_6-MBP tagged proteins (91 kDa) and purified using Ni-NTA affinity chromatography. The position of mutant proteins is indicated with red arrow. As the purified mutant proteins were mixed to varied extents with bacterial chaperon proteins DnaK (70 kDa) and SlyD (25 kDa), and trace amounts of some other impurities, the samples were subjected to SDS-PAGE analysis on a BioRad Gel Reader to estimate the percentage of the mutant proteins in total proteins.

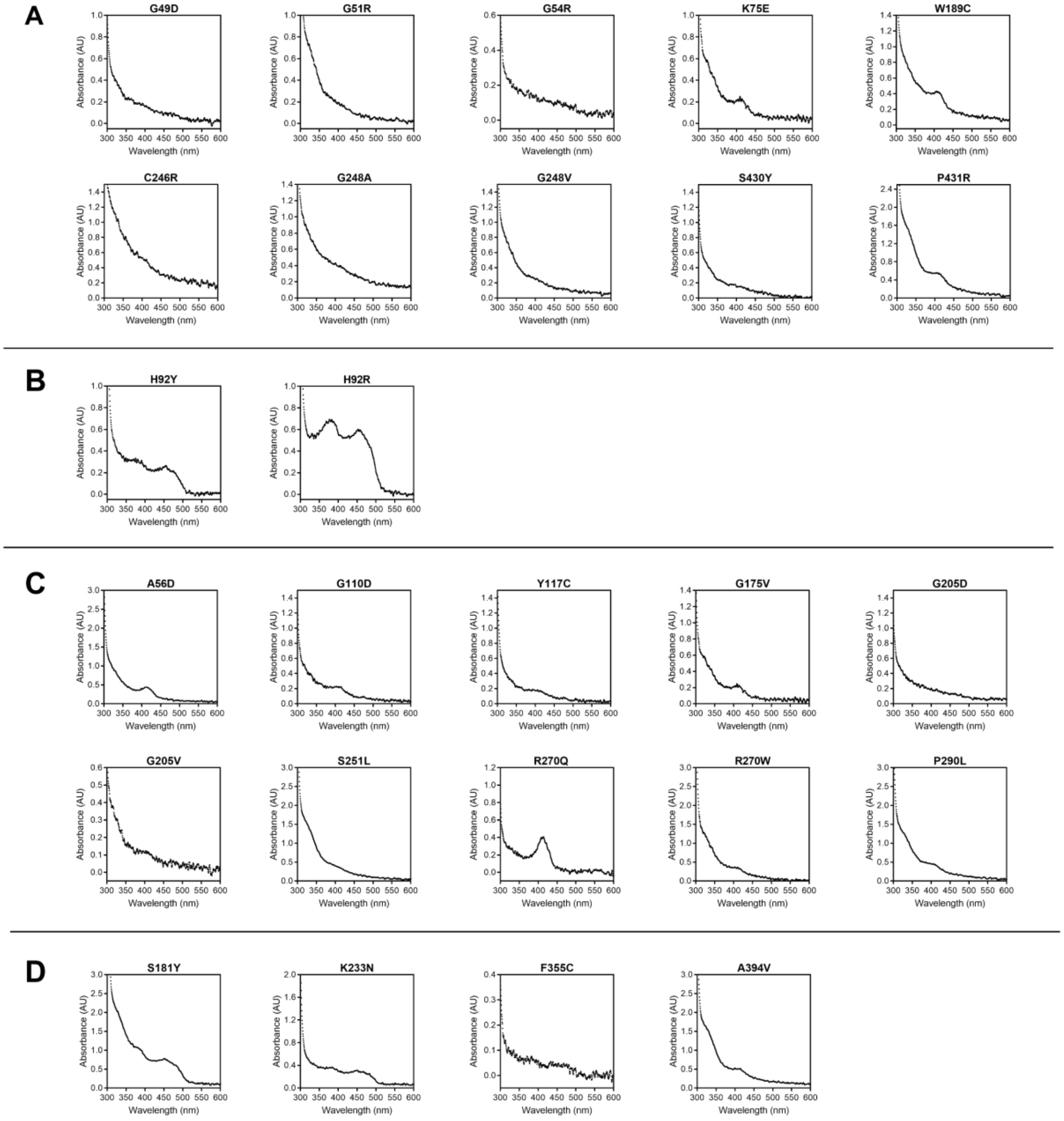

Figure S10. UV-Vis spectra of MBP-*dm*L2HGDH mutants. (A) UV-Vis spectra of MBP-*dm*L2HGDH mutants containing G49D, G51R, G54R, K75E, W189C, C246R, G248A, G248V, S430Y and P431R mutations. (B) UV-Vis spectra of MBP-*dm*L2HGDH mutants containing H92R and H92Y mutations. (C) UV-Vis spectra of MBP-d*m*L2HGDH mutants containing A56D, G110D, Y117C, G175V, G205D, G205V, S251L, R270Q, R270W and P290L mutations. (D) UV-Vis spectra of MBP-d*m*L2HGDH mutants containing S181Y, K233N, F355C, and A394V mutations.

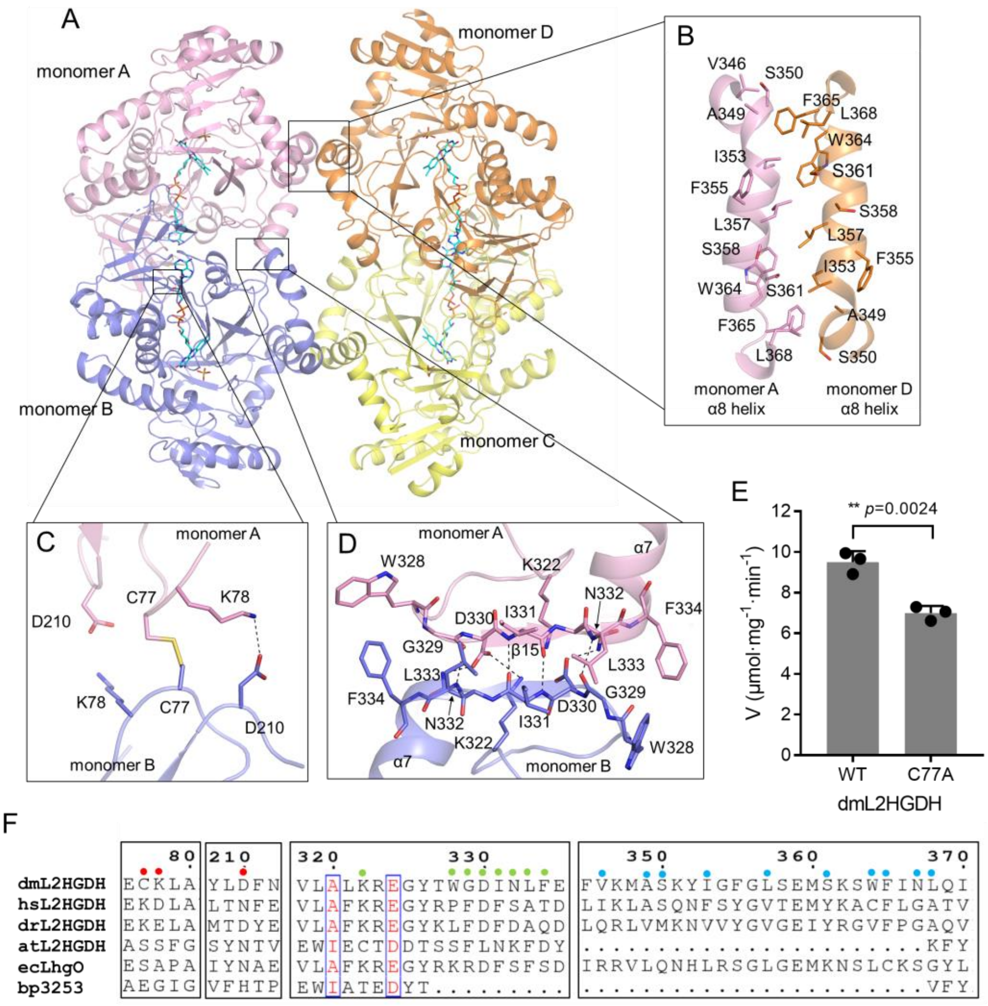

Figure S11. Assembly of the *dm*L2HGDH$^{FAD+SO4}$ tetramer. (A) Overall structure of the *dm*L2HGDH$^{FAD+SO4}$ tetramer. There are two types of dimer interfaces: dimer interface AB or CD, and dimer interface AD or BC. The interacting regions at the dimer interfaces are marked with black rectangles. FAD and SO_4^{2-} are shown with stick models and colored in cyan. **(B)** Zoom-in view of the interactions mediated by α8 helices at the dimer interface between monomers A and D. **(C)** Zoom-in view of the disulfide bond between Cys77 residues at the dimer interface between monomers A and B. **(D)** Zoom-in view of the interactions mediated by the β15 strands at the dimer interface between monomers A and B. Residues involved in the interactions are shown with stick models, and are colored in the same scheme as the relevant monomer. **(E)** The specific activity of WT *dm*L2HGDH and C77A mutant using L-2-HG (1.5 mM) as substrate. The error bars represent the standard deviations of three independent experiments. *P* value was calculated using two-sided unpaired t test. **(F)** Sequence alignment of the regions involved in the formations of the dimer interfaces. The residues involved in the interactions in panels B, C, and D are indicated with red, green, and cyan spheres, respectively.

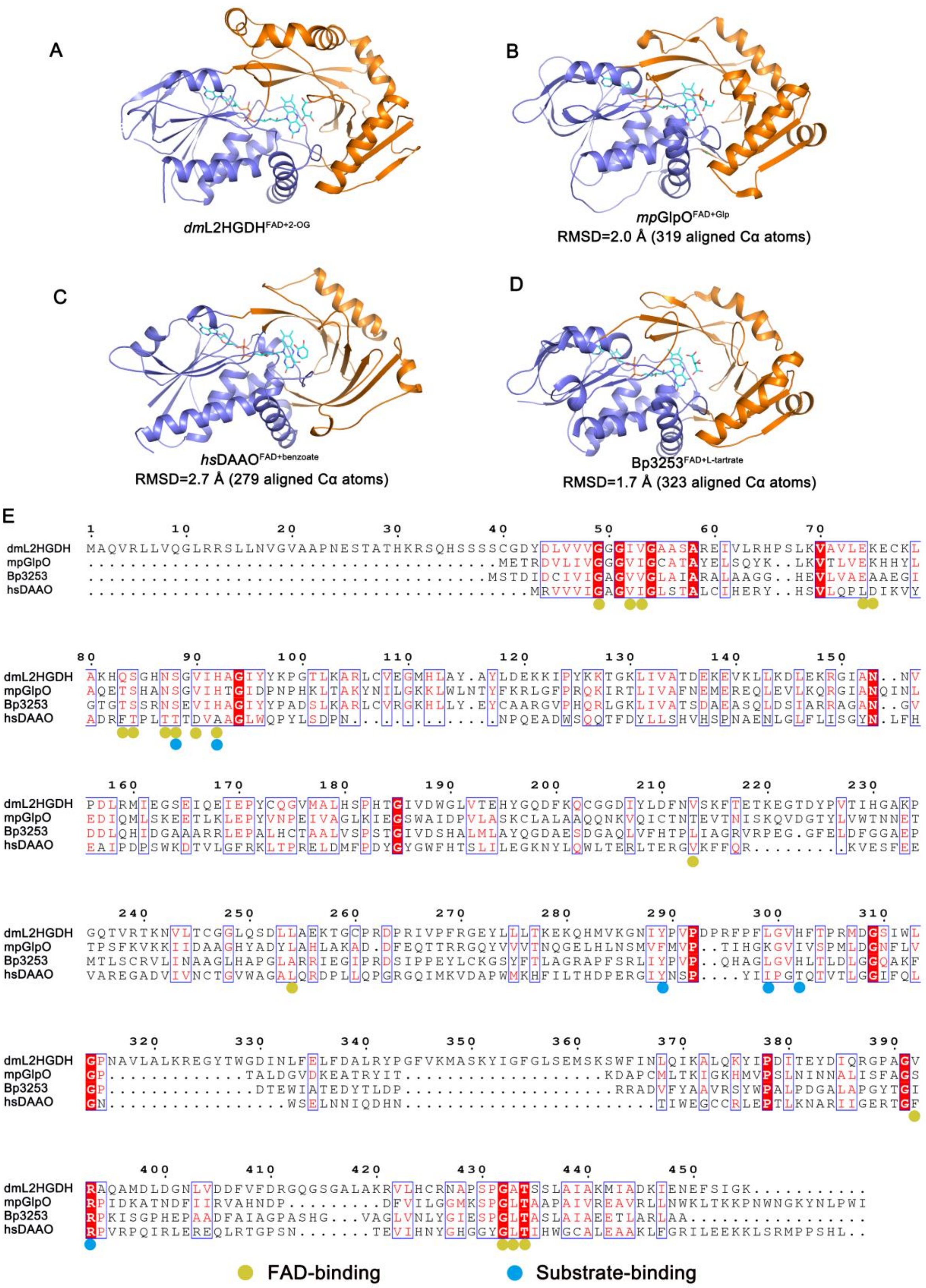
A
dmL2HGDH^FAD+2-OG
B
mpGlpO^FAD+Glp
RMSD=2.0 Å (319 aligned Cα atoms)
C
hsDAAO^FAD+benzoate
RMSD=2.7 Å (279 aligned Cα atoms)
D
Bp3253^FAD+L-tartrate
RMSD=1.7 Å (323 aligned Cα atoms)
E
dmL2HGDH MAQVRLLVQGLRRSLLNVGVAAPNESTATHKRSQHSSSSCGDYDLVVVGGGIVGAASAREIVLRHPSLKVAVLEKECKL
mpGlpO ....METRDVLIVGGGVIGCATAYELSQYK..LKVTLVEKHHYL
Bp3253 ......................................MSTDIDCIVIGAGVVGLAIARALAAGG..HEVLVAEAAEGI
hsDAAO ....MRVVVIGAGVIGLSTALCIHERY..HSVLQPLDIKVY
dmL2HGDH AKHQSGHNSGVIHAGIYYKPGTLKARLCVEGMHLAY.AYLDEKKIPYKKTGKLIVATDEKEVKLLKDLEKRGIAN..NV
mpGlpO AQETSHANSGVIHTGIDPNPHKLTAKYNILGKKLWLNTYFKRLGFPRQKIRTLIVAFNEMEREQLEVLKQRGIANQINL
Bp3253 GTGTSSRNSEVIHAGIYYPADSLKARLCVRGKHLLY.EYCAARGVPHQRLGKLIVATSDAEASQLDSIARRAGAN..GV
hsDAAO ADRFTPLTTTDVAAGLWQPYLSDPN............NPQEADWSQQTFDYLLSHVHSPNAENLGLFLISGYN.LFH
dmL2HGDH PDLRMIEGSEIQEIEPYCQGVMALHSPHTGIVDWGLVTEHYGQDFKQCGGDIYLDFNVSKFTETKEGTDYPVTIHGAKP
mpGlpO EDIQMLSKEETLKLEPYVNPEIVAGLKIEGSWAIDPVLASKCLALAAQQNKVQICTNTEVTNISKQVDGTYLVWTNNET
Bp3253 DDLQHIDGAAARRLEPALHCTAALVSPSTGIVDSHALMLAYQGDAESDGAQLVFHTPLIAGRVRPEG.GFELDFGGAEP
hsDAAO EAIPDPSWKDTVLGFRKLTPRELDMFPDYGYGWFHTSLILEGKNYLQWLTERLTERGVKFFQR........KVESFEE
dmL2HGDH GQTVRTKNVLTCGGLQSDLLAEKTGCPRDPRIVPFRGEYLLLTKEKQHMVKGNIYPVPDPRFPFLGVHFTPRMDGSIWL
mpGlpO TPSFKVKKIIDAAGHYADYLAHLAKAD.DFEQTTRRGQYVVVTNQGELHLNSMVFMVP..TIHGKGVIVSPMLDGNFLV
Bp3253 MTLSCRVLINAAGLHAPGLARRIEGIPRDSIPPEYLCKGSYFTLAGRAPFSRLIYPVP..QHAGLGVHLTLDLGGQAKF
hsDAAO VAREGADVIVNCTGVWAGALQRDPLLQPGRGQIMKVDAPWMKHFILTHDPERGIYNSP....YIIPGTQTVTLGGIFQL
dmL2HGDH GPNAVLALKREGYTWGDINLFELFDALRYPGFVKMASKYIGFGLSEMSKSWFINLQIKALQKYIPDITEYDIQRGPAGV
mpGlpO GP.............TALDGVDKEATRYIT...................KDAPCMLTKIGKHMVPSLNINNALISFAGS
Bp3253 GP.............DTEWIATEDYTLDP...................RRADVFYAAVRSYWPALPDGALAPGYTGI
hsDAAO GN................WSELNNIQDHN........................TIWEGCCRLEPTLKNARIIGERTGF
dmL2HGDH RAQAMDLDGNLVDDFVFDRGQGSGALAKRVLHCRNAPSPGATSSLAIAKMIADKIENEFSIGK...........
mpGlpO RPIDKATNDFIIRVAHNDP.........DFVILGGMKSPGLTAAPAIVREAVRLLNWKLTKKPNWNGKYNLPWI
Bp3253 RPKISGPHEPAADFAIAGPASHG...VAGLVNLYGIESPGLTASLAIAEETLARLAA.................
hsDAAO RPVRPQIRLEREQLRTGPSN.......TEVIHNYGHGGYGLTIHWGCALEAAKLFGRILEEKKLSRMPPSHL..
FAD-binding
Substrate-binding

Figure S12. **Comparison of *dm*L2HGDH with other members of the DAAO family.** (A) Structure of *dm*L2HGDH in complex with FAD and 2-OG. (B) Structure of *M. pneumonia* GlpO (*mp*GlpO) in complex with substrate L-2-glycerophosphate (Glp, PDB 4X9M). (C) Structure of human DAAO (*hs*DAAO) in complex with inhibitor benzoate (PDB 2DU8). (D) Structure of *B. pertussis* Bp3253 in complex with L-tartrate (PDB 3DME). The bound ligands are shown with stick models. The FAD-binding domains of the proteins are colored in blue, and the substrate-binding domains are colored in orange. The RMSD values are calculated by superposition of the *mp*GlpO, *hs*DAAO and Bp3253 structures on the *dm*L2HGDH structure. (E) Sequence alignment of dmL2HGDH and other DAAO family members. The key residues of *dm*L2HGDH involved in the binding of 2-OG and FAD are indicated with cyan and yellow spheres, respectively.

Table S1. Functional characterization of mutations of *dm*L2HGDH corresponding to L-2-HGA-associated *hs*L2HGDH mutations.

*hs*L2HGDH mutation	Corresponding *dm*L2HGDH mutation	Residual activity of *dm*L2HGDH mutant (%)	Location on *dm*L2HGDH structure	Pathogenicity prediction (score)[b]
**Substrate-binding site**				
H98R (67)	H92R	ND[a]	β2-α2 loop	Damaging (1.000)
H98Y (68,69)	H92Y	ND	β2-α2 loop	Damaging (1.000)
**FAD-binding site**				
G55D (68,70)	G49D	ND	β1-α1 loop	Damaging (1.000)
G57R (67,71,72)	G51R	ND	β1-α1 loop	Damaging (1.000)
G60R (73)	G54R	ND	α1	Damaging (1.000)
K81E (37,53,74,85)	K75E	ND	β2-α2 loop	Damaging (0.993)
Y195C (70,75)	W189C	ND	α5	Damaging (0.988)
C258R (75)	C246R	ND	β10-α6 loop	Damaging (1.000)
G260A (55,76)	G248A	ND	β10-α6 loop	Damaging (1.000)
G260V (55)	G248V	ND	β10-α6 loop	Damaging (1.000)
S440Y (70,72)	S430Y	ND	β18-α11 loop	Damaging (1.000)
P441R (55)	P431R	ND	β18-α11 loop	Damaging (1.000)
**Others**				
A62D (77,85)	A56D	ND	α1	Damaging (0.998)
G116D (55)	G110D	ND	α2	Damaging (1.000)
Y123C (78)	Y117C	ND	α2	Damaging (0.999)
K136R (79)	K130R	No expression	β3	Damaging (1.000)
A140P (55)	A134P	No expression	β3-α3 loop	Damaging (1.000)
G156V (70)	G150V	ND	α3	Damaging (0.996)
E176G (55)	E170G	No expression	α4-β5 loop	Damaging (0.991)
E176D (37,53)	E170D	No expression	α4-β5 loop	Damaging (0.995)
C179R (55)	C173R	ND	α4-β5 loop	Damaging (0.993)
G181V (80)	G175V	ND	α4-β5 loop	Damaging (1.000)
A184V (55)	A178V	No expression	β5	Damaging (0.996)
C187Y (54)	S181Y	13.2	β5-α5 loop	Damaging (0.996)

G211D (55)	G205D	ND	α4-β6 loop	Possibly damaging (0.493)
G211V (71,81)	G205V	ND	α4-β6 loop	Possibly damaging (0.889)
K246N (55)	K233N	26.2	β8-β9 loop	Benign (0.014)
S263L (70)	S251L	ND	α6	Damaging (0.992)
R282Q (82,83,84)	R270Q	ND	β11	Damaging (1.000)
R282W (55)	R270W	ND	β11	Damaging (1.000)
V296E (69)	V284E	No expression	η1-β12 loop	Damaging (0.964)
P302L (68,70)	P290L	ND	β12-β13 loop	Damaging (0.999)
E336K (55)	E324K	No expression	β14-β15 loop	Damaging (0.976)
Y367C (55)	F355C	16.9	α8	Damaging (0.930)
A406V (55)	A394V	3.3	β16	Possibly damaging (0.873)
H434P (67)	H424P	No expression	β18	Damaging (0.997)

[a] ND, not detectable.

[b] Pathogenicity was predicted using PolyPhen-2 server (58). The score in the bracket indicates the degree of pathogenicity from benign (score = 0) to damaging (score = 1).
